# Supplementary figures and images for: “Gap hunting” to characterize clustered probe signals in Illumina methylation array data
Source: Epigenetics Chromatin. 2016 Dec 7;9:56. doi: 10.1186/s13072-016-0107-z (PMC5142147; doi:10.1186/s13072-016-0107-z)

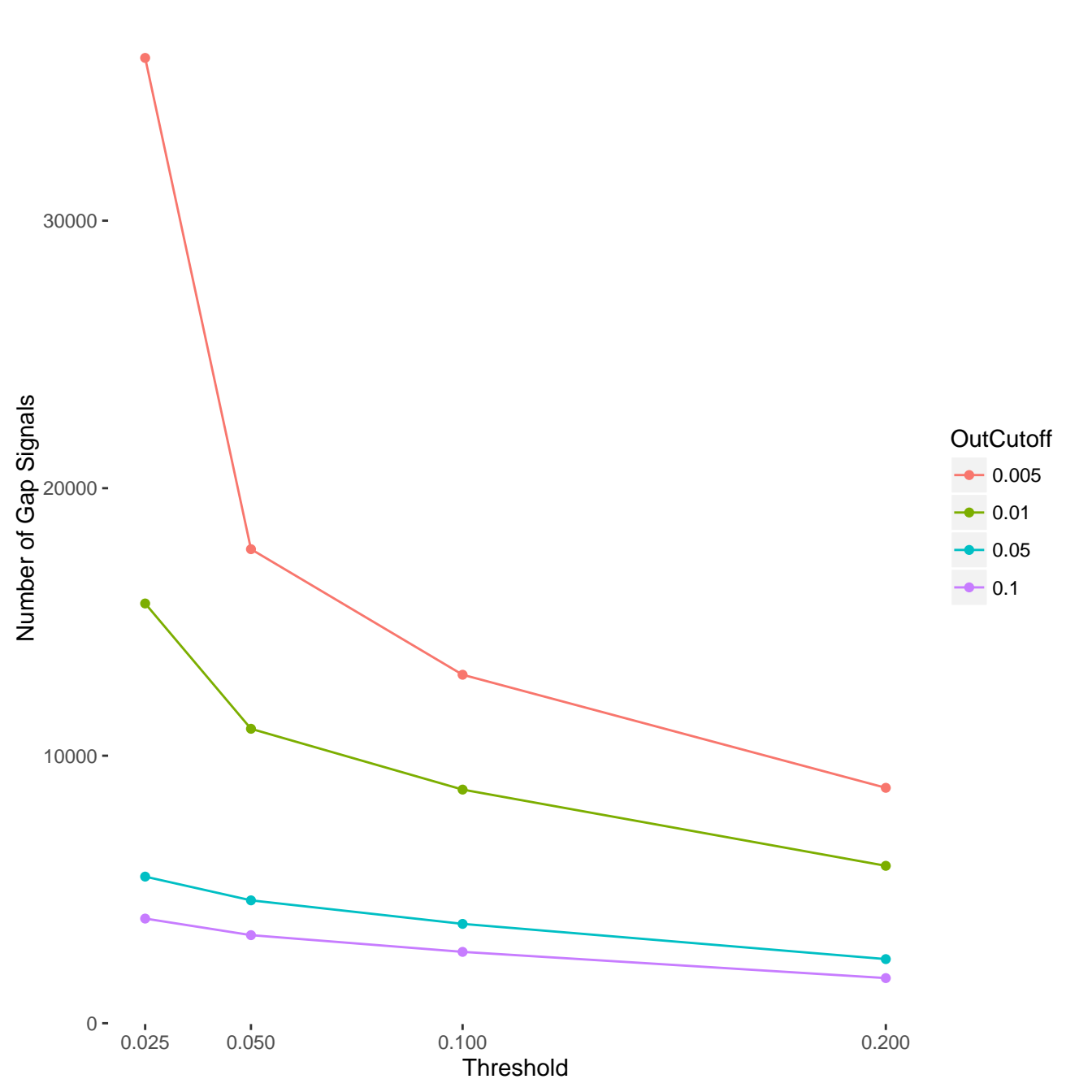

Supplement: Supplementary file 1 — Additional file 1: Figure S1. Number of gap signals detected in SEED at various combinations of the “threshold” and “outCutoff” arguments to gaphunter(). [file 13072_2016_107_MOESM1_ESM.pdf]

A

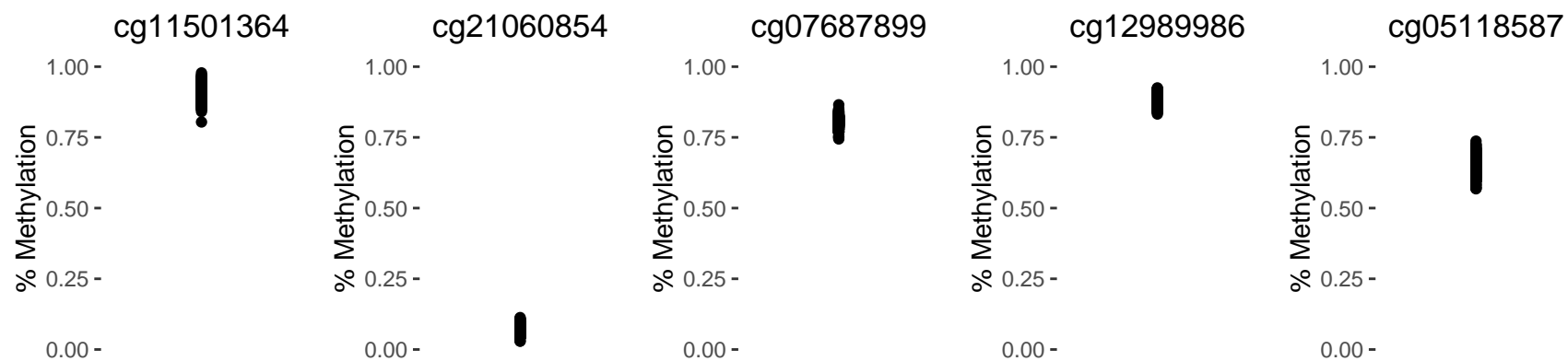

B

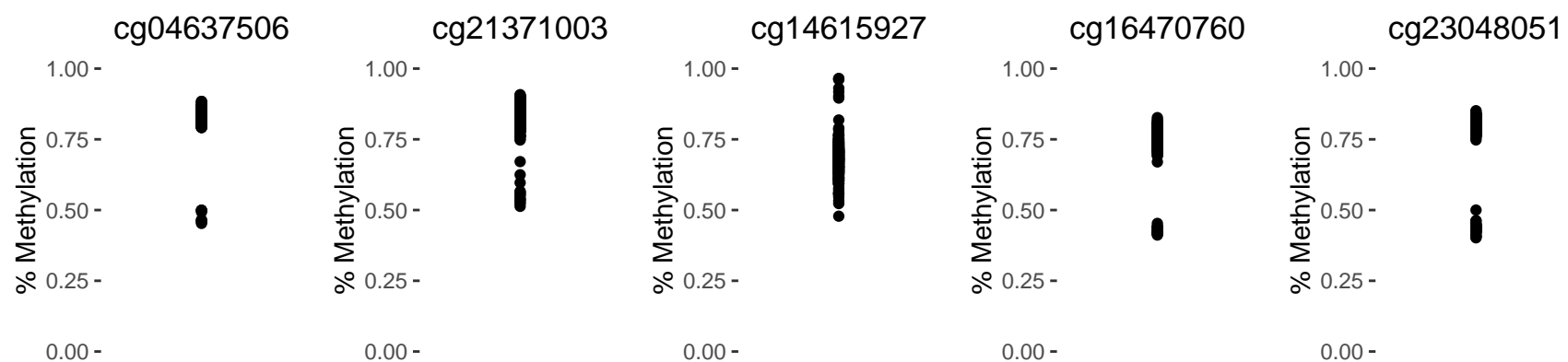

C

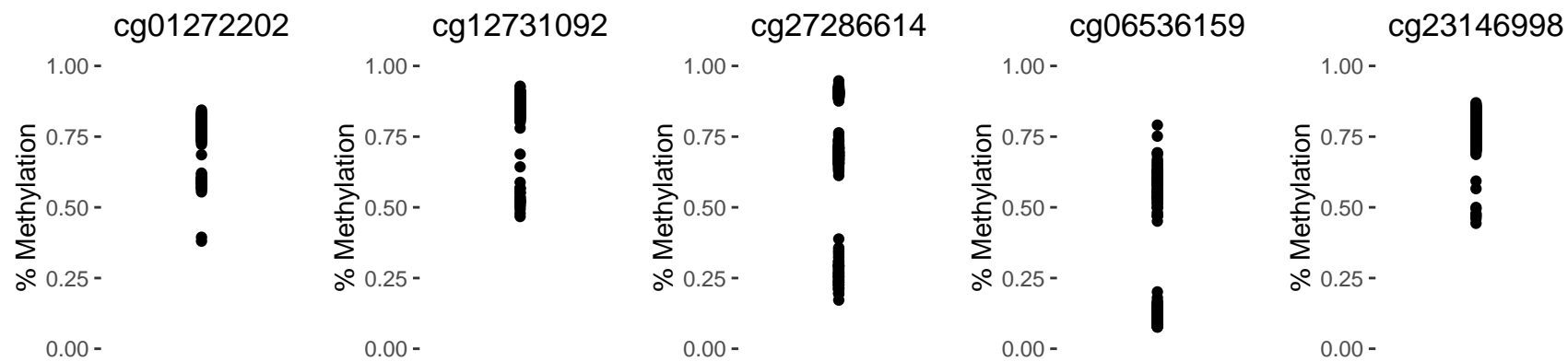

Supplement: Supplementary file 2 — Additional file 2: Figure S2. Examples of non-gap and gap signals found in SEED at 5% “threshold” argument and 1% “outCutoff” argument. a 5 probes not identified as gap signals. b 5 probes identified as gap signals with 2 clusters. c 5 probes identified as gap signals with 3 clusters. [file 13072_2016_107_MOESM2_ESM.pdf]

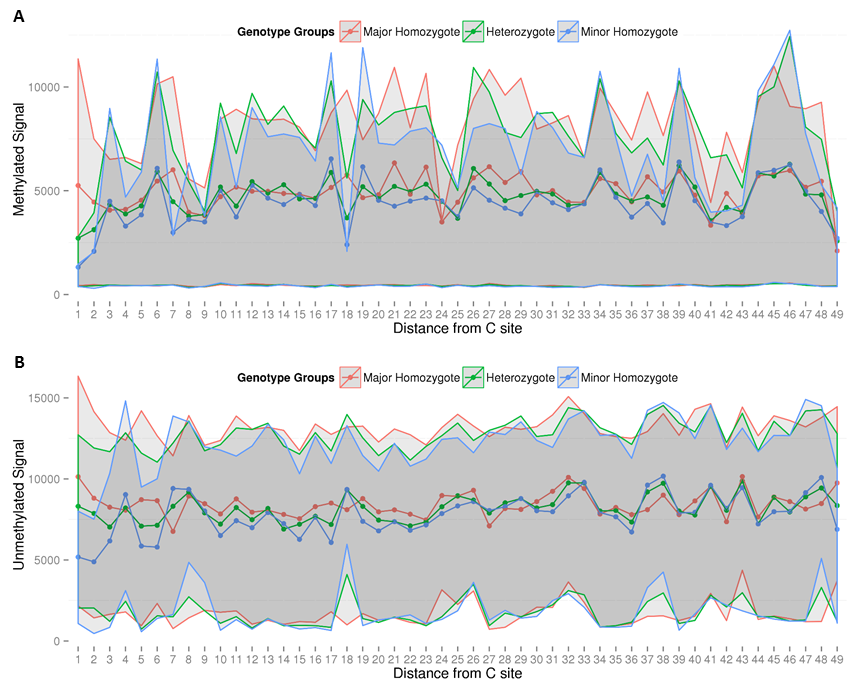

Supplement: Supplementary file 7 — Additional file 7: Figure S32. The effect of SNPs located in Type I probes outside of the CpG or SBE position on methylated signal and unmethylated signal. We examined specific scenarios in which the following conditions were met: a probe contained a measured SNP in the 50 bp probe length, it also did not contain a SNP mapping to the C, G and/or SBE sites, and it contained only a single SNP in the probe length. We found all probes that met this criteria and varying values of distance from the SNP to the measured C site (1–50 bp). At each distance value, we plotted the mean and inter-quartile range of the people who were homozygous for the reference allele (“Major Homozygote”), heterozygous (“Heterozygote”) or homozygous for the minor allele (“Minor Homozygote”). The degree of overlap between these 3 lines and their respective IQRs therefore demonstrates the effect of a polymorphism on subsequent 450k signal; the lack of overlap is directly correlated to an increased influence of the: polymorphism. For both methylated signal (a) and unmethylated signal (b), polymorphisms at closer distance to the C site drive discordance between the 3 genotype groups. The relationship is less clear than for Type II probes, most likely because there are fewer Type I probes generally (and further fewer in this specific scenario) and the Type I design assumes that CpG sites within the probe length match that the methylation state of the interrogated CpG site. This assumption would be violated given our inclusion criteria for this analysis if the polymorphisms in question here occur at the C site of CpG site within the 50 bp probe length. [file 13072_2016_107_MOESM7_ESM.png]

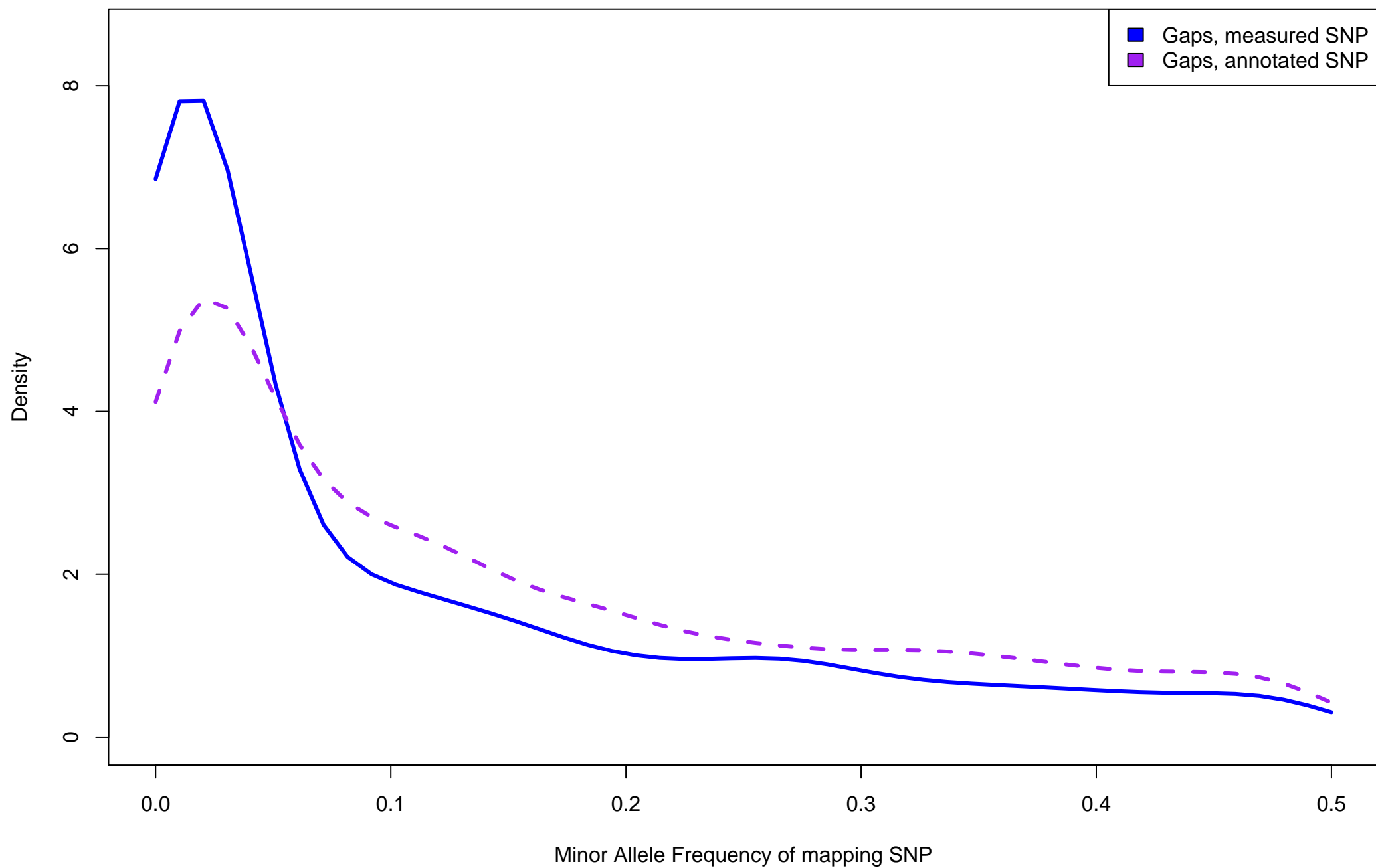

Supplement: Supplementary file 8 — Additional file 8: Figure S33. MAF distributions of measured SNPs vs annotated SNPs that map to 450k probes. We calculated the minor allele frequency (MAF) of all measured SNPs that mapped to gap signals, and determined the MAF for all of the annotated SNPs that map to gap signals as seen in the dbSNP138 annotation. The greater amount of SNPs with high MAF (>0.1) in the annotated SNP group may account for the higher area under the curve at higher standard deviation values as seen in Fig. 8. [file 13072_2016_107_MOESM8_ESM.pdf]

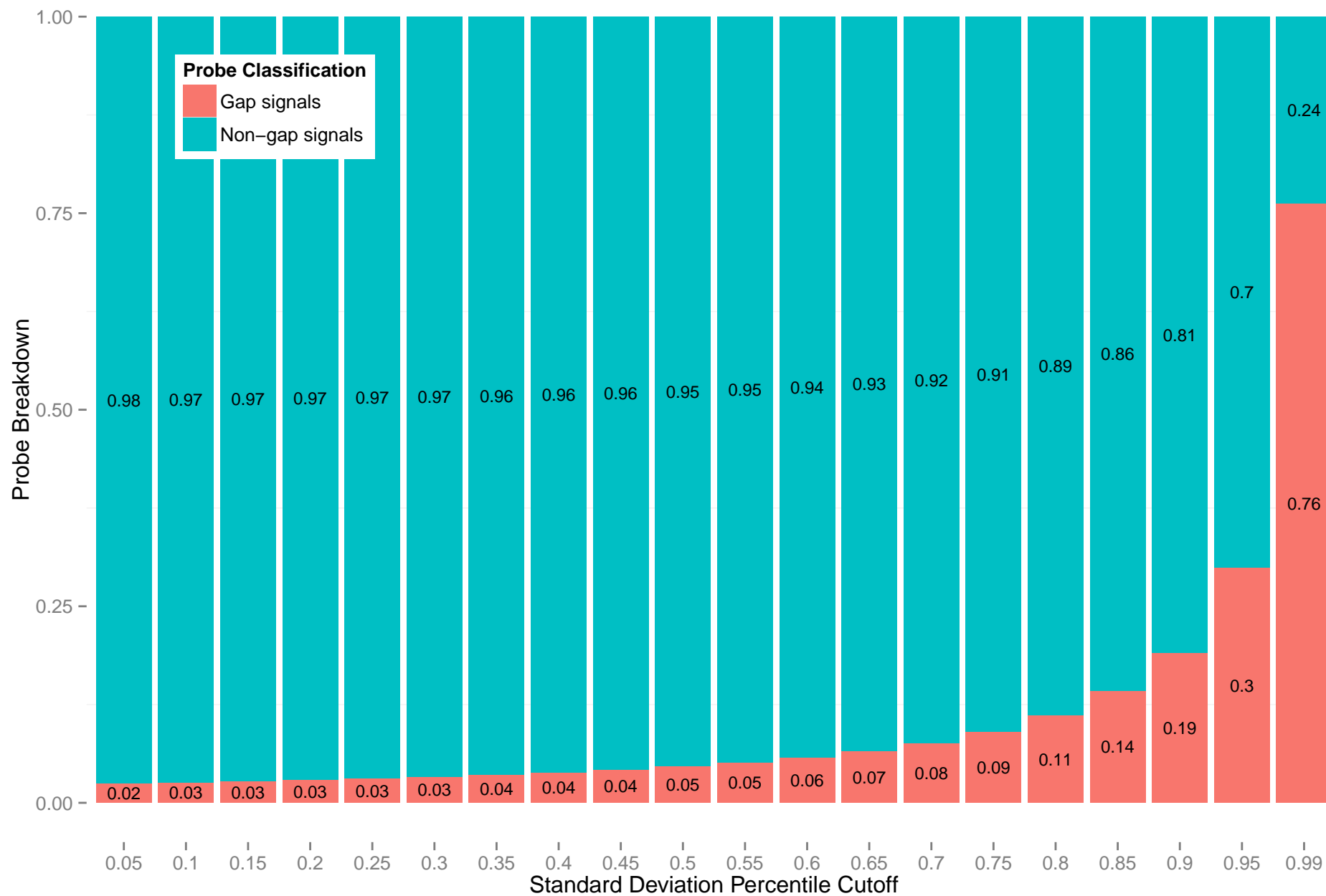

Supplement: Supplementary file 11 — Additional file 11: Figure S34. Filtering on variably methylated probes at various cutoffs in the context of gap signals. We calculated the proportion of gap and non-gap signals at various percentile thresholds of standard deviation cutoff (1–99%) to define a variably methylated probe. Researchers who filter on variable methylation prior to association analysis should be cautioned to be increasingly aware of gap signals (and subsequently their implications on DNAm related to disease described herein) as the cutoff to define a variably methylated probe increases. [file 13072_2016_107_MOESM11_ESM.pdf]
