# Supplementary material for: “Gap hunting” to characterize clustered probe signals in Illumina methylation array data
Source: Epigenetics Chromatin. 2016 Dec 7;9:56. doi: 10.1186/s13072-016-0107-z (PMC5142147; doi:10.1186/s13072-016-0107-z)
Supplement: Supplementary file 5 — Additional file 5: Figures S3–S25. All Remaining C and G site scenarios for Type II and Type I probes. Each additional scenario of a C and G site-mapping SNP delimited in Fig. 2 not including the scenario show in Fig. 3. Each of these figures contains the same panels (a–d) as seen in Fig. 3. All scenarios demonstrate the expected behavior shown in Fig. 2. [file 13072_2016_107_MOESM5_ESM.pdf]

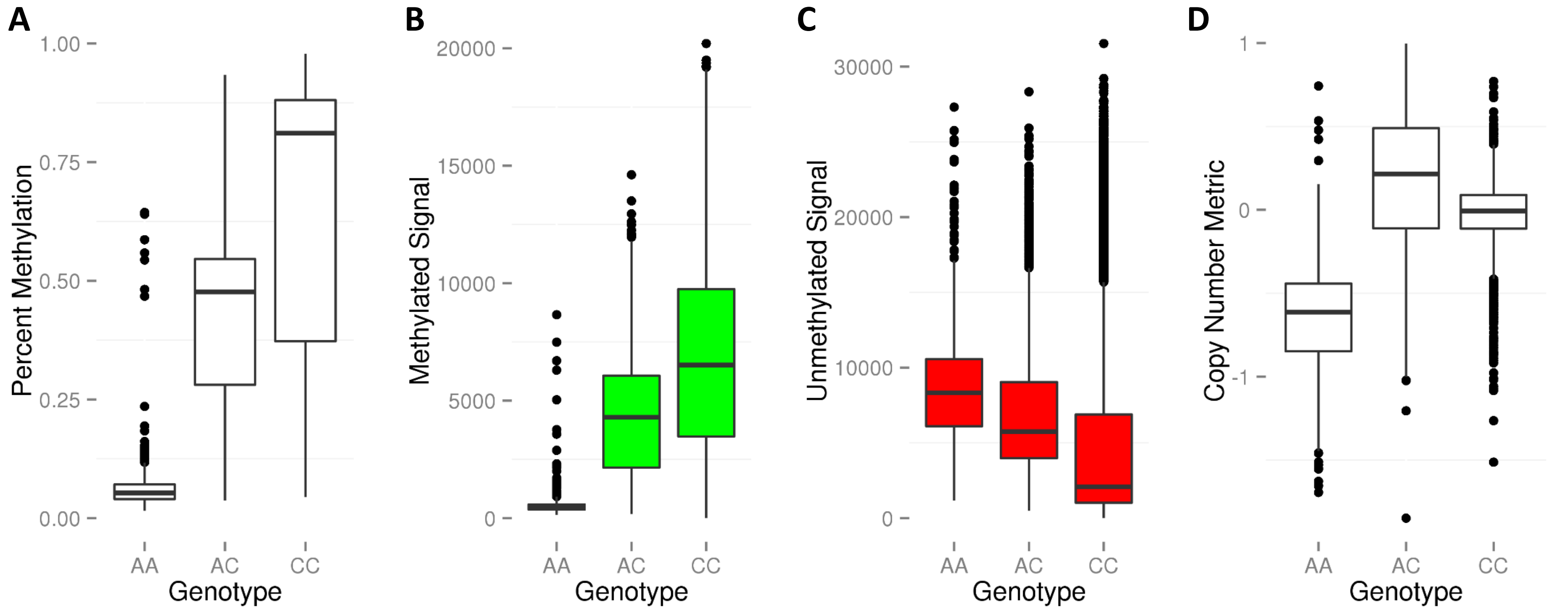

Figure S3: The effect of a A/C SNP at a forward strand C site of Type II 450k probes on percent methylation, methylated signal, unmethylated signal, and a copy number metric. 84 probes in SEED were classified in this specific scenario. **Panel A**: Percent methylation (beta value) vs. genotype. **Panel B**: Methylated signal vs genotype. **Panel C**: Unmethylated signal vs genotype. **Panel D**: Copy number metric (see Methods for calculation) vs. genotype.

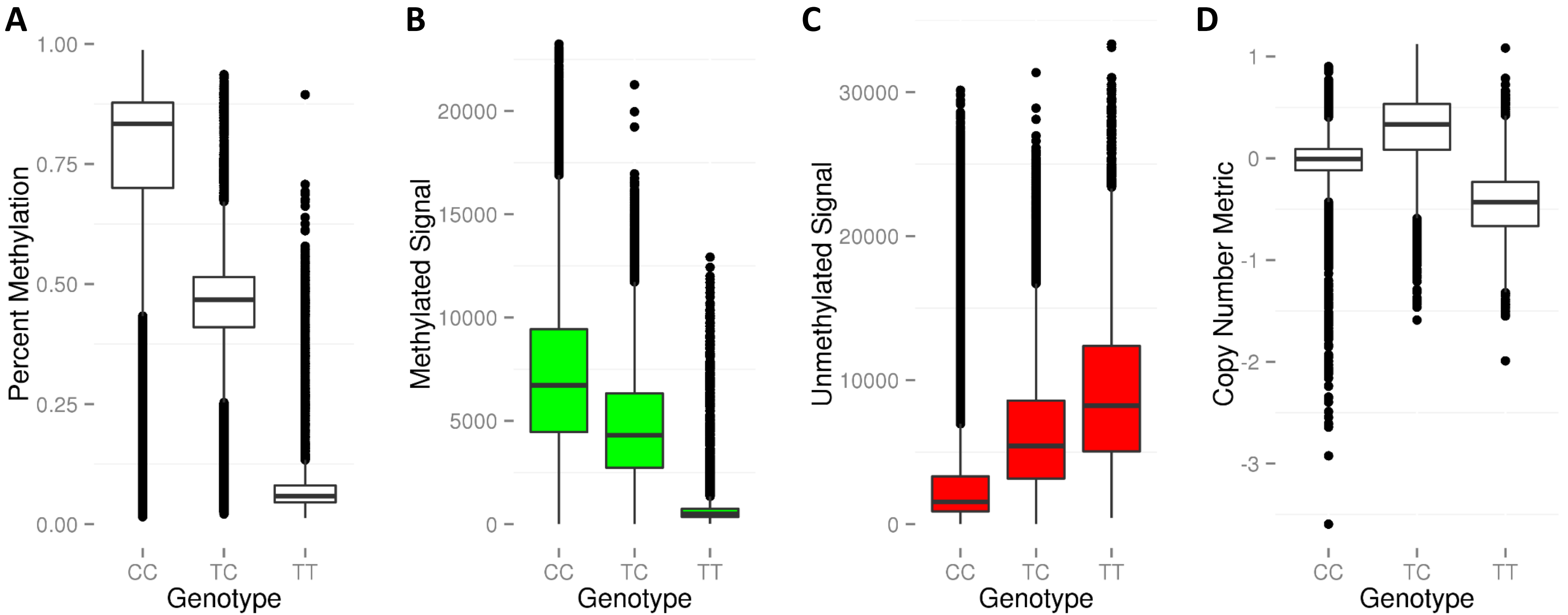

Figure S4: The effect of a T/C SNP at a forward strand C site of Type II 450k probes on percent methylation, methylated signal, unmethylated signal, and a copy number metric. 824 probes in SEED were classified in this specific scenario. **Panel A**: Percent methylation (beta value) vs. genotype. **Panel B**: Methylated signal vs genotype. **Panel C**: Unmethylated signal vs genotype. **Panel D**: Copy number metric (see Methods for calculation) vs. genotype.

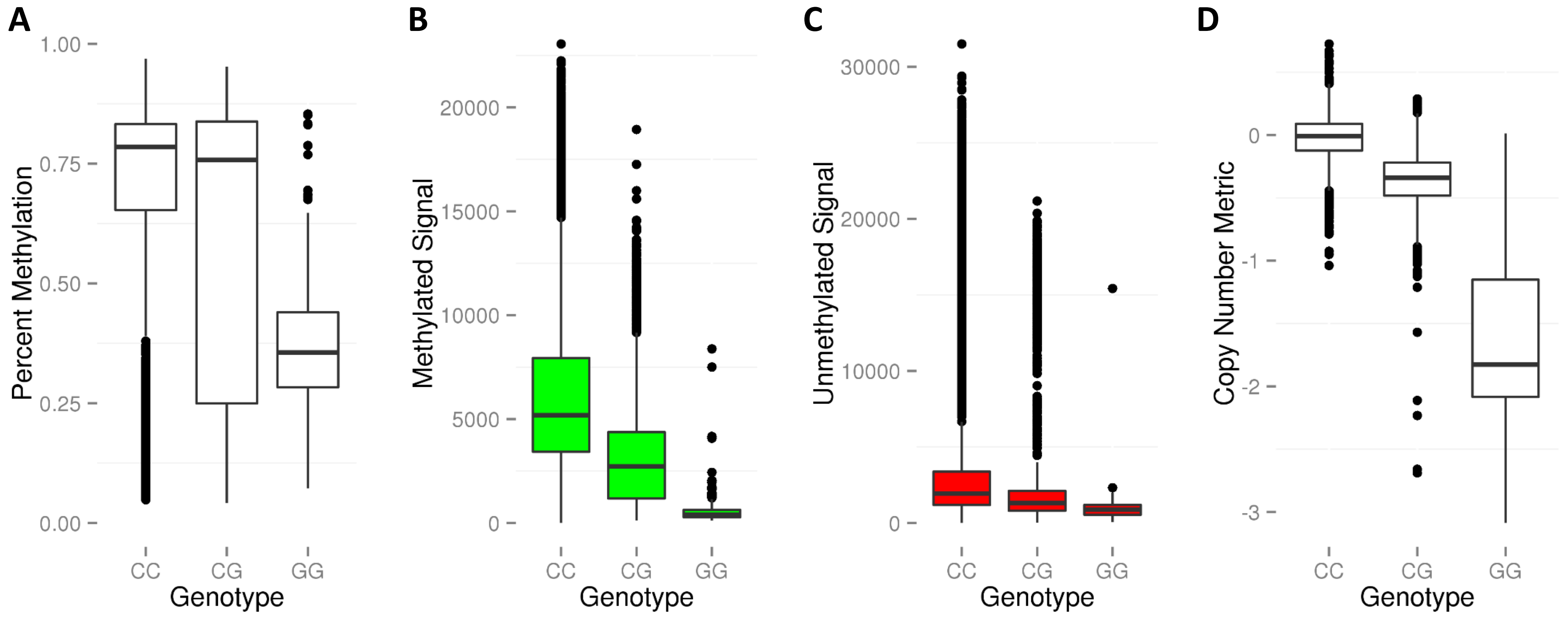

Figure S5: The effect of a C/G SNP at a reverse strand C site of Type II 450k probes on percent methylation, methylated signal, unmethylated signal, and a copy number metric. 60 probes in SEED were classified in this specific scenario. **Panel A**: Percent methylation (beta value) vs. genotype. **Panel B**: Methylated signal vs genotype. **Panel C**: Unmethylated signal vs genotype. **Panel D**: Copy number metric (see Methods for calculation) vs. genotype.

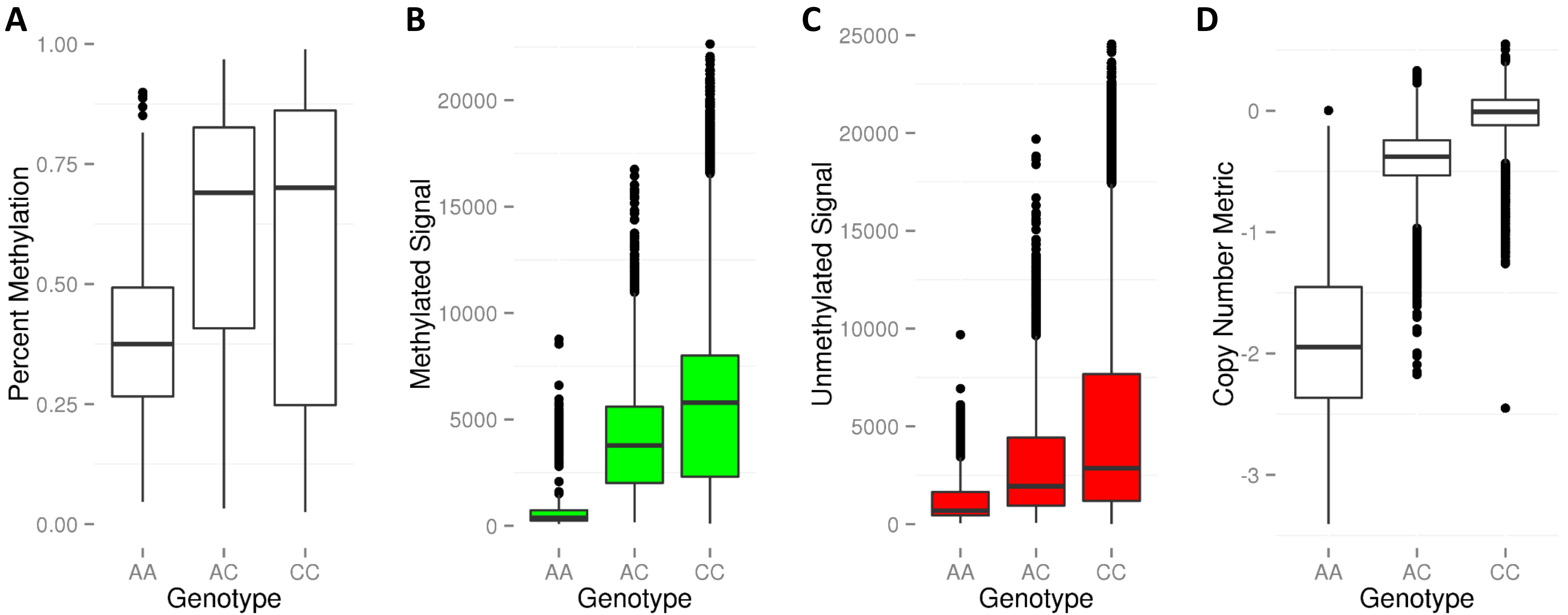

Figure S6: The effect of a A/C SNP at a reverse strand C site of Type II 450k probes on percent methylation, methylated signal, unmethylated signal, and a copy number metric. 76 probes in SEED were classified in this specific scenario. **Panel A**: Percent methylation (beta value) vs. genotype. **Panel B**: Methylated signal vs genotype. **Panel C**: Unmethylated signal vs genotype. **Panel D**: Copy number metric (see Methods for calculation) vs. genotype.

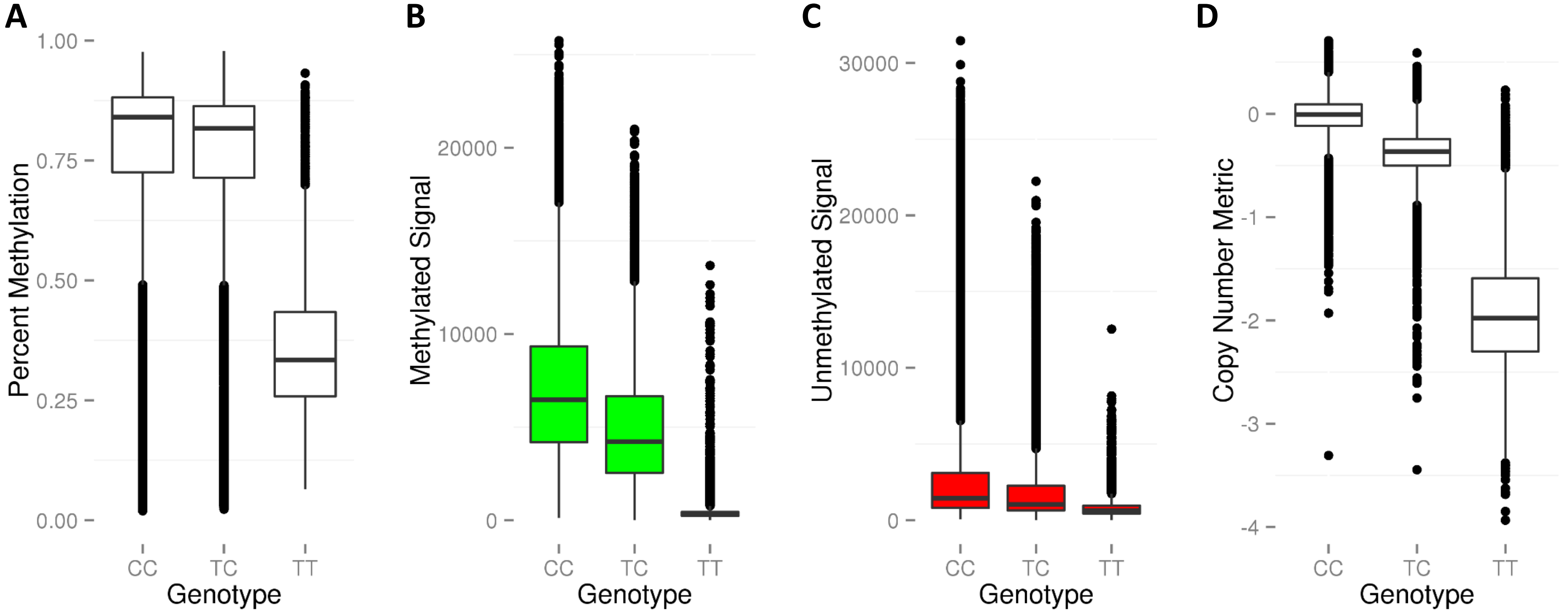

Figure S7: The effect of a T/C SNP at a reverse strand C site of Type II 450k probes on percent methylation, methylated signal, unmethylated signal, and a copy number metric. 775 probes in SEED were classified in this specific scenario. **Panel A**: Percent methylation (beta value) vs. genotype. **Panel B**: Methylated signal vs genotype. **Panel C**: Unmethylated signal vs genotype. **Panel D**: Copy number metric (see Methods for calculation) vs. genotype.

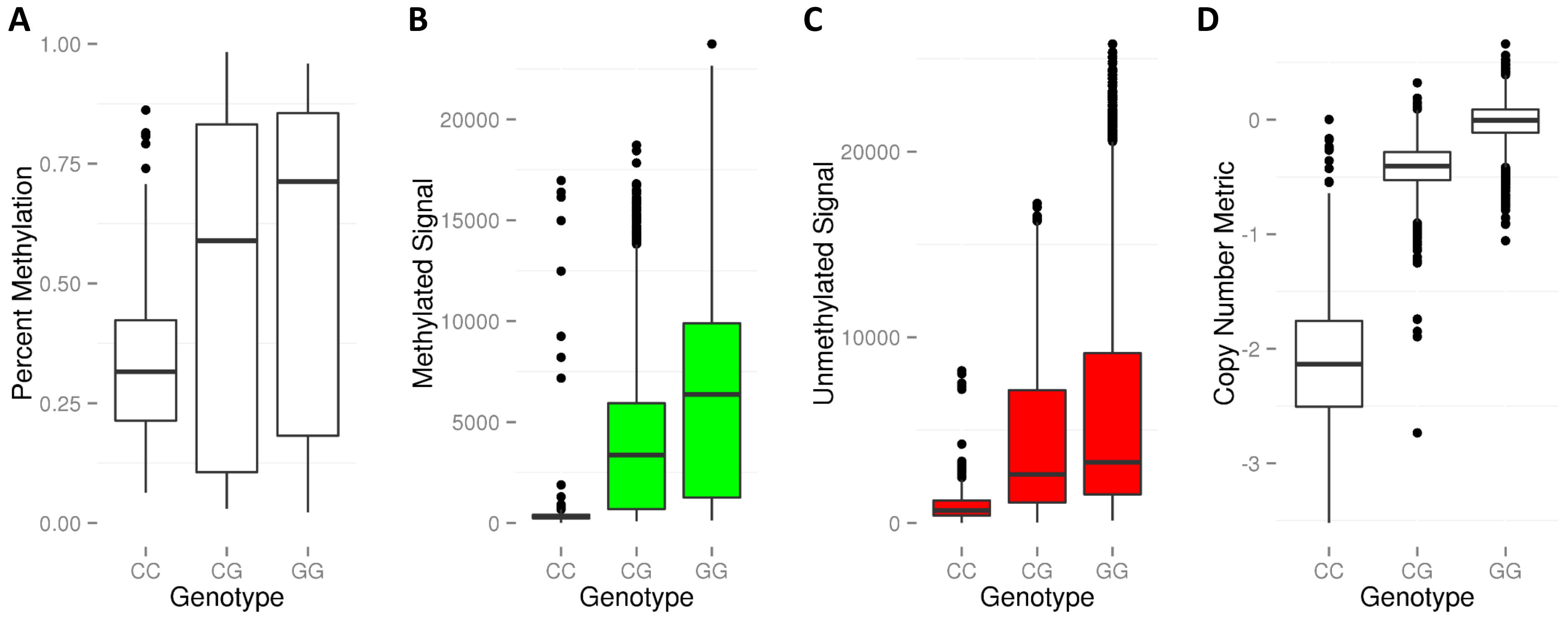

Figure S8: The effect of a C/G SNP at a forward strand G site of Type II 450k probes on percent methylation, methylated signal, unmethylated signal, and a copy number metric. 67 probes in SEED were classified in this specific scenario. **Panel A**: Percent methylation (beta value) vs. genotype. **Panel B**: Methylated signal vs genotype. **Panel C**: Unmethylated signal vs genotype. **Panel D**: Copy number metric (see Methods for calculation) vs. genotype.

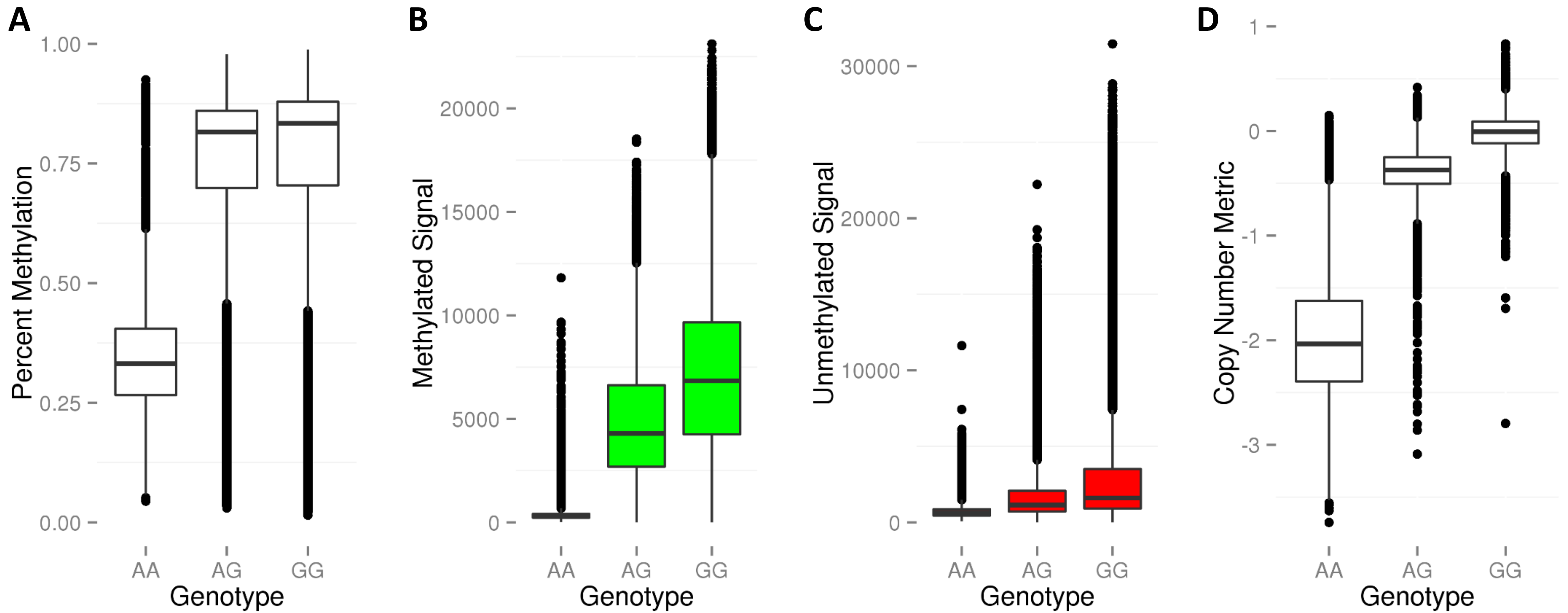

Figure S9: The effect of a A/G SNP at a forward strand G site of Type II 450k probes on percent methylation, methylated signal, unmethylated signal, and a copy number metric. 826 probes in SEED were classified in this specific scenario. **Panel A**: Percent methylation (beta value) vs. genotype. **Panel B**: Methylated signal vs genotype. **Panel C**: Unmethylated signal vs genotype. **Panel D**: Copy number metric (see Methods for calculation) vs. genotype.

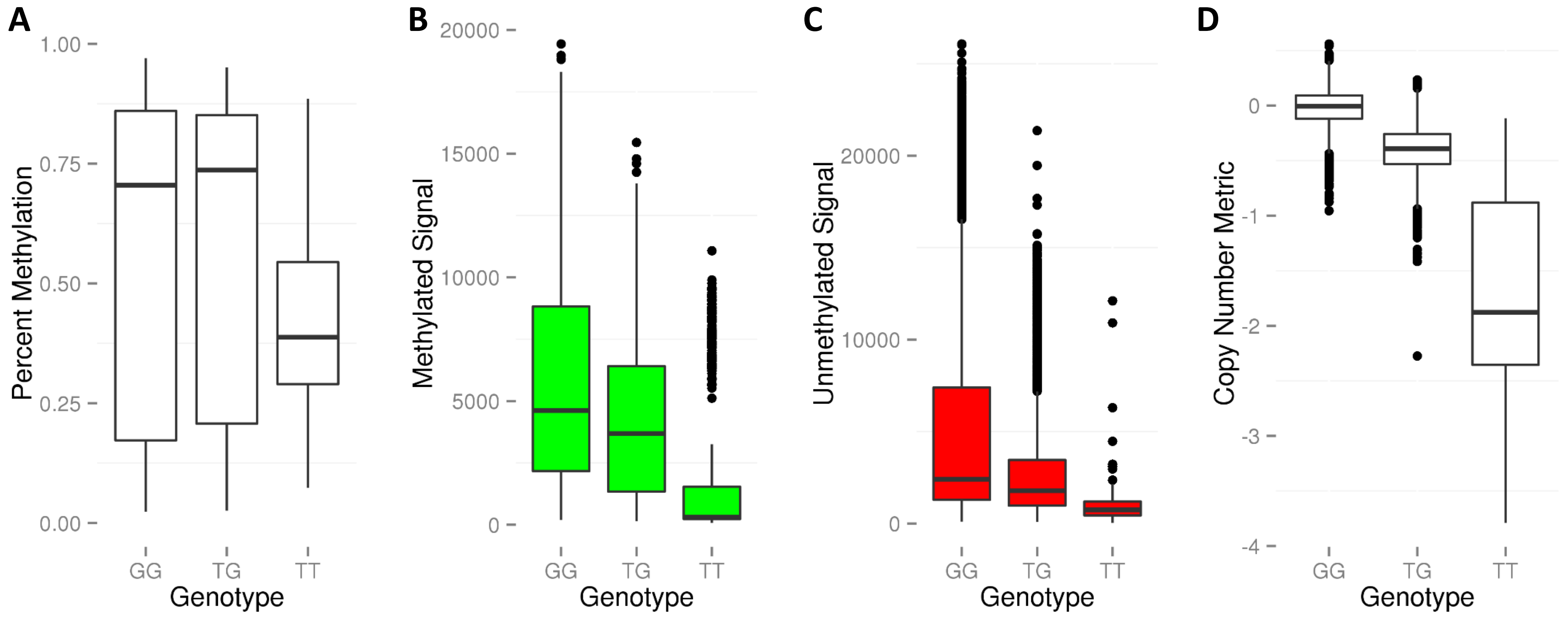

Figure S10: The effect of a T/G SNP at a forward strand G site of Type II 450k probes on percent methylation, methylated signal, unmethylated signal, and a copy number metric. 67 probes in SEED were classified in this specific scenario. **Panel A**: Percent methylation (beta value) vs. genotype. **Panel B**: Methylated signal vs genotype. **Panel C**: Unmethylated signal vs genotype. **Panel D**: Copy number metric (see Methods for calculation) vs. genotype.

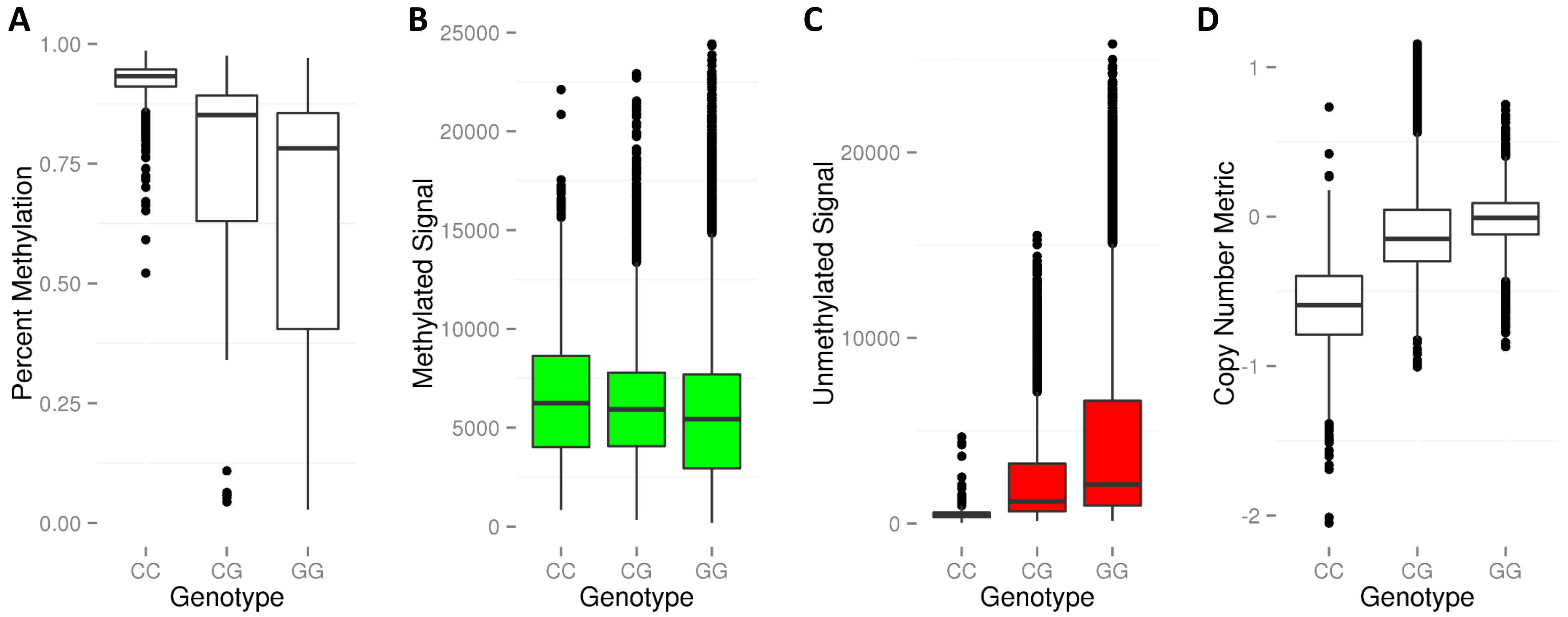

Figure S11: The effect of a C/G SNP at a negative strand G site of Type II 450k probes on percent methylation, methylated signal, unmethylated signal, and a copy number metric. 95 probes in SEED were classified in this specific scenario. **Panel A**: Percent methylation (beta value) vs. genotype. **Panel B**: Methylated signal vs genotype. **Panel C**: Unmethylated signal vs genotype. **Panel D**: Copy number metric (see Methods for calculation) vs. genotype.

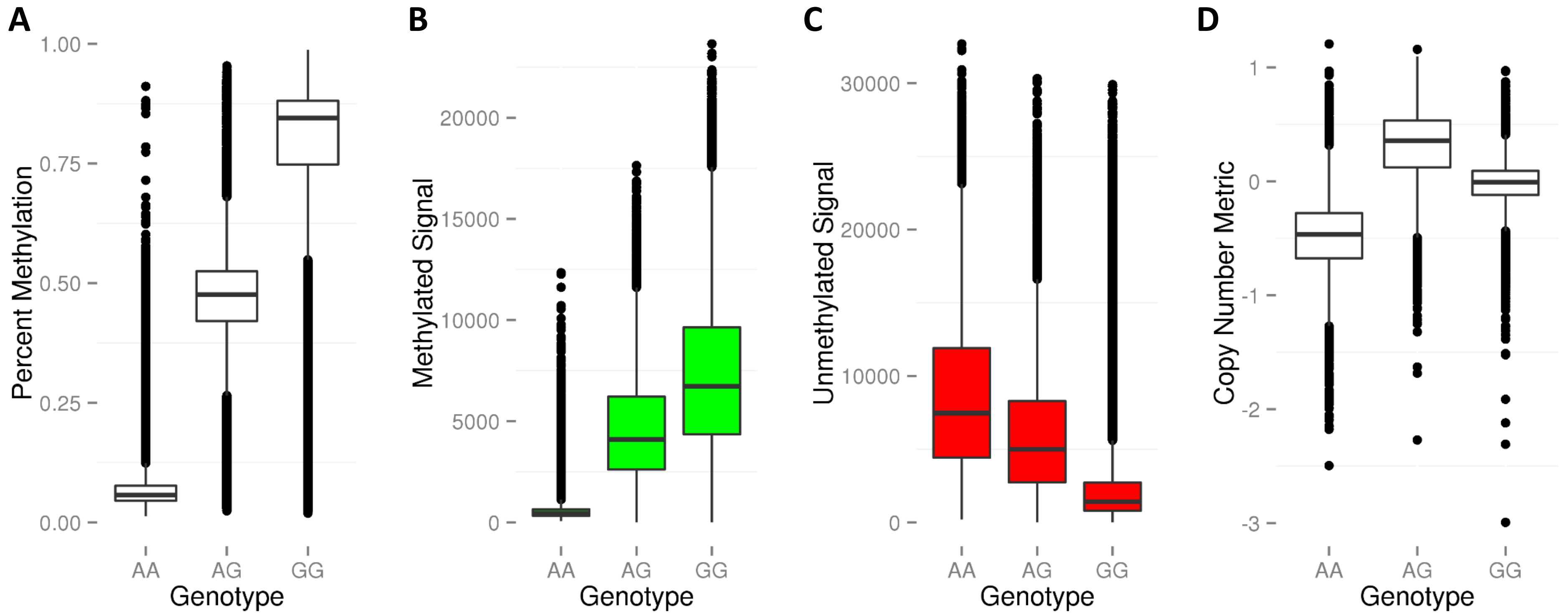

Figure S12: The effect of a A/G SNP at a negative strand G site of Type II 450k probes on percent methylation, methylated signal, unmethylated signal, and a copy number metric. 1258 probes in SEED were classified in this specific scenario. **Panel A**: Percent methylation (beta value) vs. genotype. **Panel B**: Methylated signal vs genotype. **Panel C**: Unmethylated signal vs genotype. **Panel D**: Copy number metric (see Methods for calculation) vs. genotype.

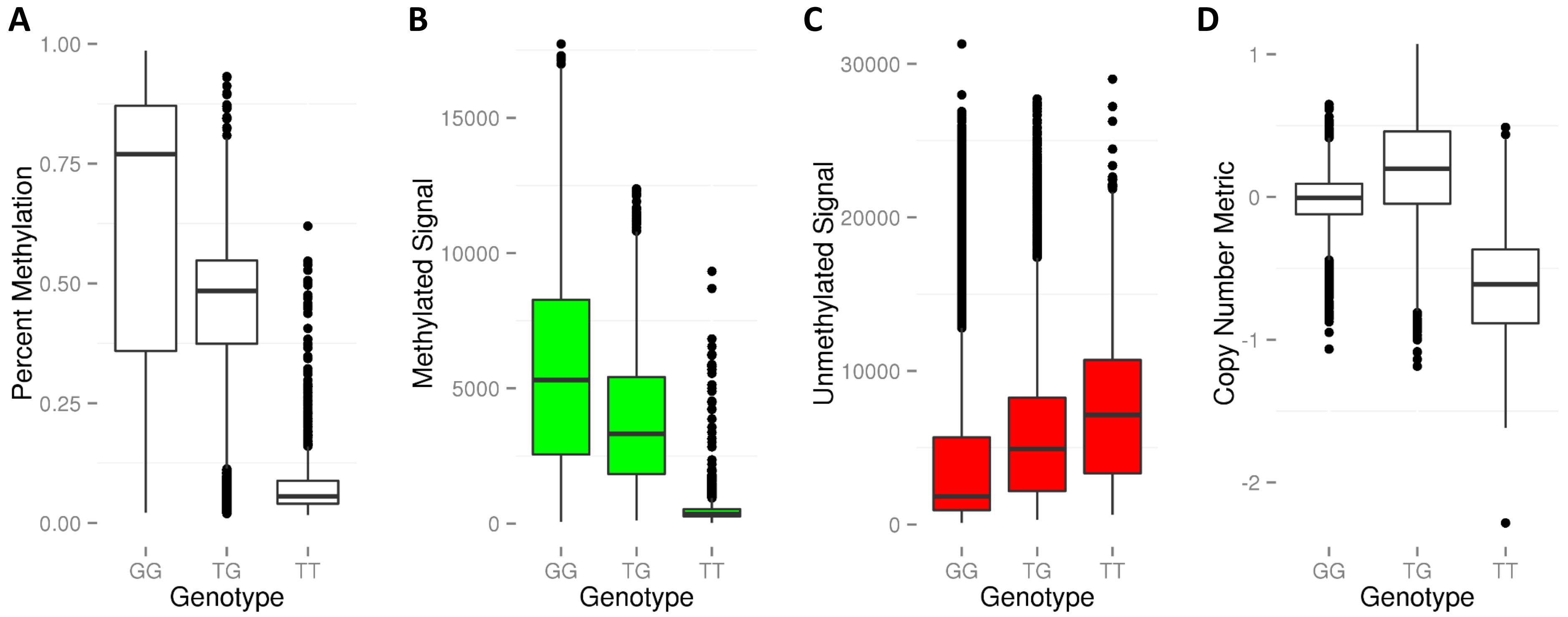

Figure S13: The effect of a T/G SNP at a negative strand G site of Type II 450k probes on percent methylation, methylated signal, unmethylated signal, and a copy number metric. 131 probes in SEED were classified in this specific scenario. **Panel A**: Percent methylation (beta value) vs. genotype. **Panel B**: Methylated signal vs genotype. **Panel C**: Unmethylated signal vs genotype. **Panel D**: Copy number metric (see Methods for calculation) vs. genotype.

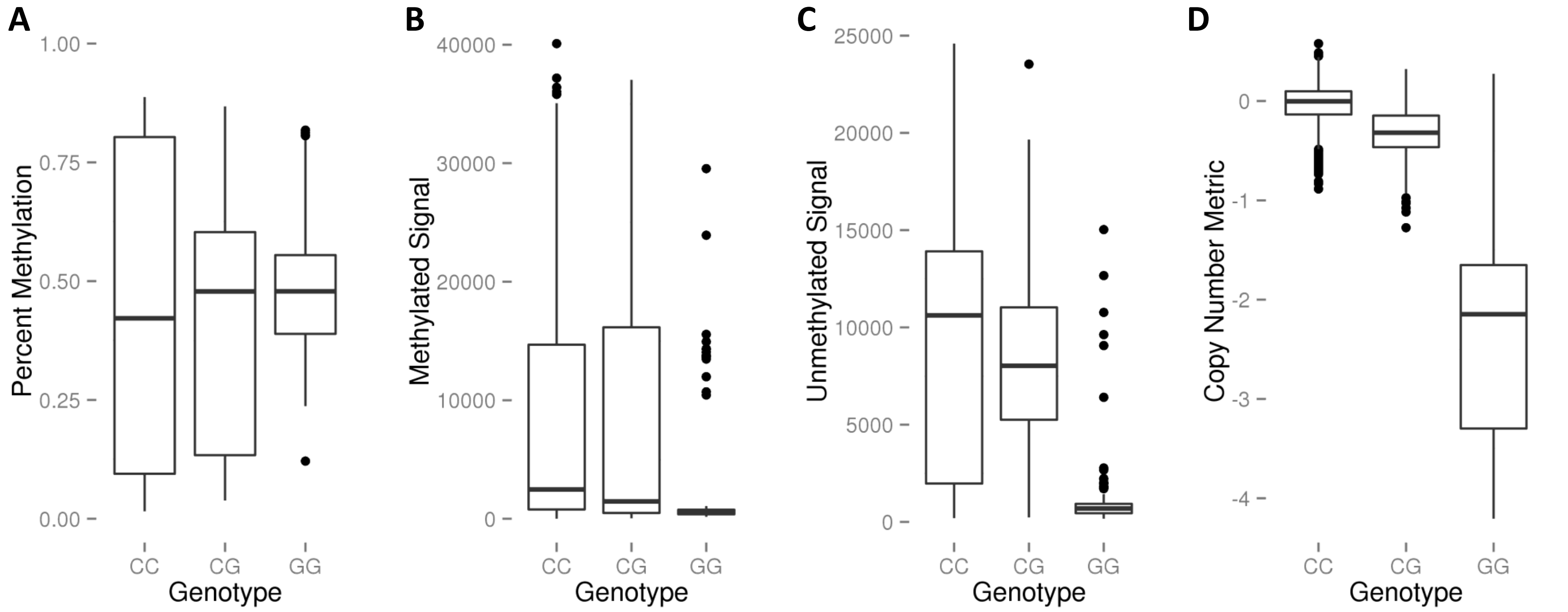

Figure S14: The effect of a C/G SNP at a positive strand C site of Type I 450k probes on percent methylation, methylated signal, unmethylated signal, and a copy number metric. 23 probes in SEED were classified in this specific scenario. **Panel A**: Percent methylation (beta value) vs. genotype. **Panel B**: Methylated signal vs genotype. **Panel C**: Unmethylated signal vs genotype. **Panel D**: Copy number metric (see Methods for calculation) vs. genotype.

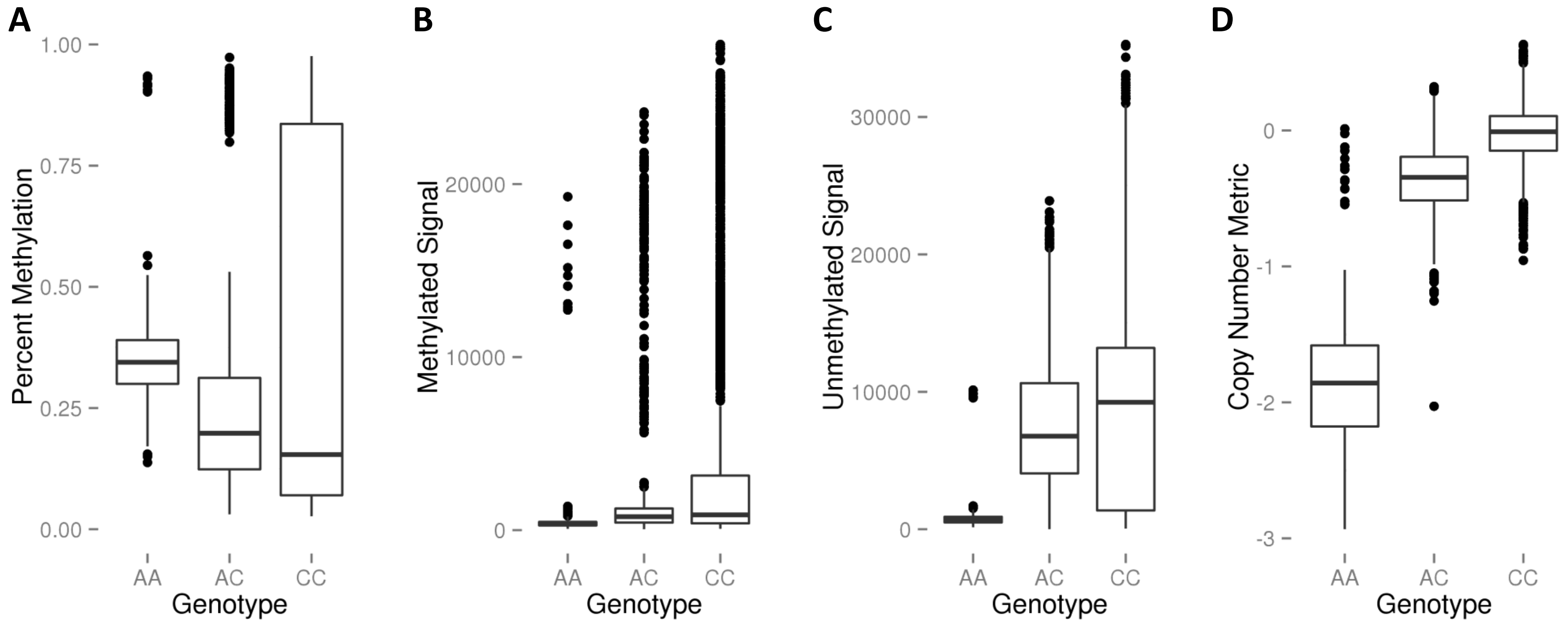

Figure S15: The effect of a A/C SNP at a positive strand C site of Type I 450k probes on percent methylation, methylated signal, unmethylated signal, and a copy number metric. 30 probes in SEED were classified in this specific scenario. **Panel A**: Percent methylation (beta value) vs. genotype. **Panel B**: Methylated signal vs genotype. **Panel C**: Unmethylated signal vs genotype. **Panel D**: Copy number metric (see Methods for calculation) vs. genotype.

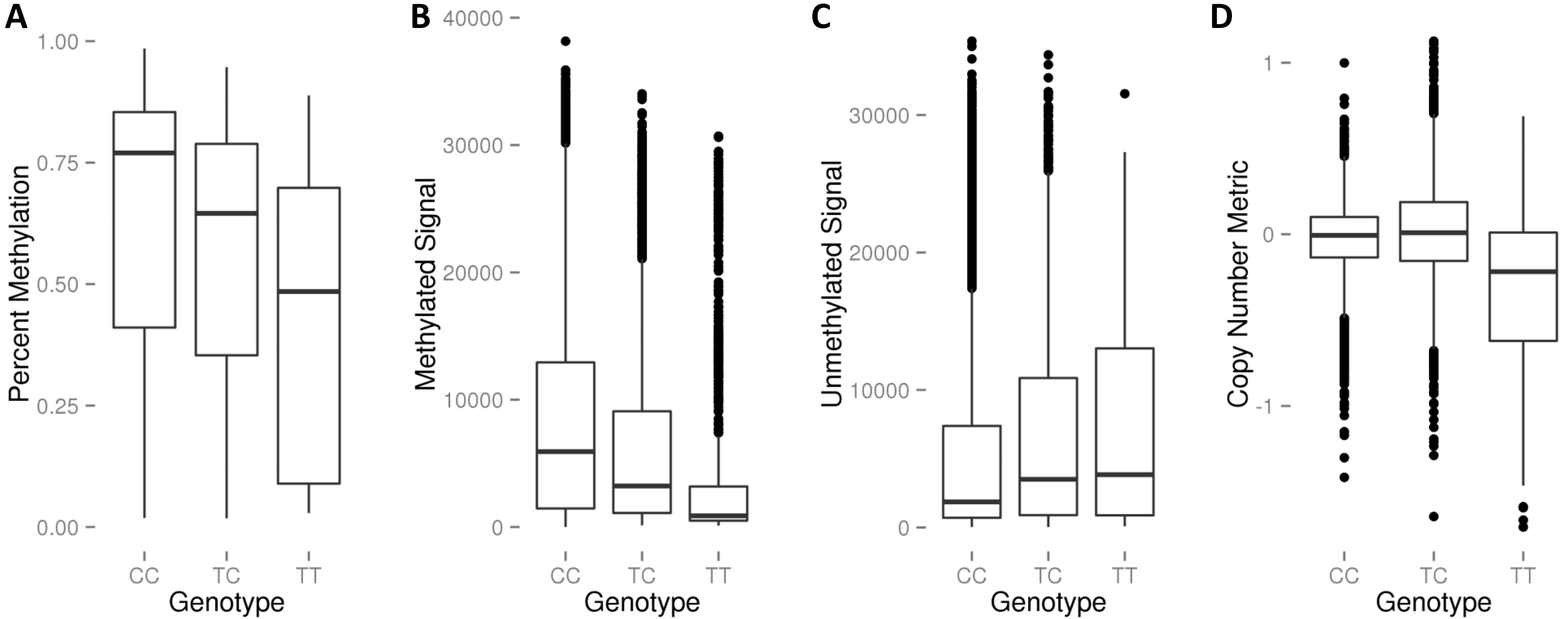

Figure S16: The effect of a T/C SNP at a positive strand C site of Type I 450k probes on percent methylation, methylated signal, unmethylated signal, and a copy number metric. 138 probes in SEED were classified in this specific scenario. **Panel A**: Percent methylation (beta value) vs. genotype. **Panel B**: Methylated signal vs genotype. **Panel C**: Unmethylated signal vs genotype. **Panel D**: Copy number metric (see Methods for calculation) vs. genotype.

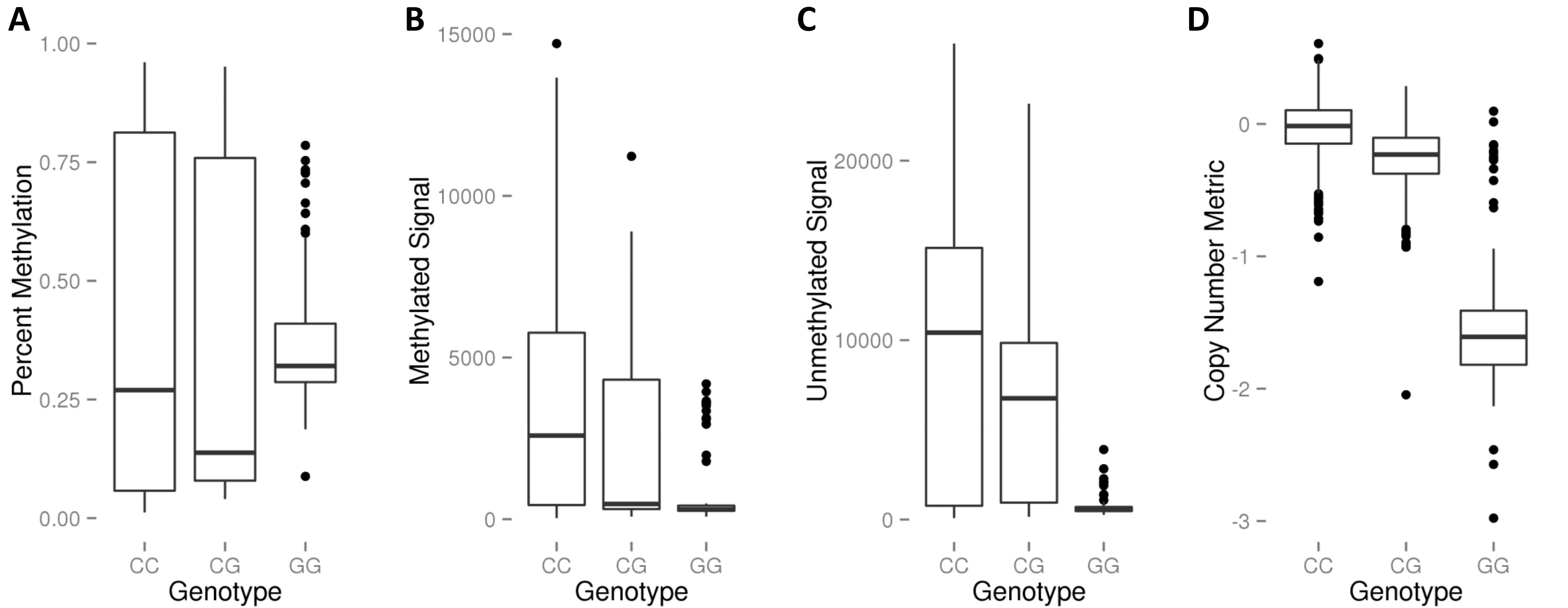

Figure S17: The effect of a C/G SNP at a negative strand C site of Type I 450k probes on percent methylation, methylated signal, unmethylated signal, and a copy number metric. 20 probes in SEED were classified in this specific scenario. **Panel A**: Percent methylation (beta value) vs. genotype. **Panel B**: Methylated signal vs genotype. **Panel C**: Unmethylated signal vs genotype. **Panel D**: Copy number metric (see Methods for calculation) vs. genotype.

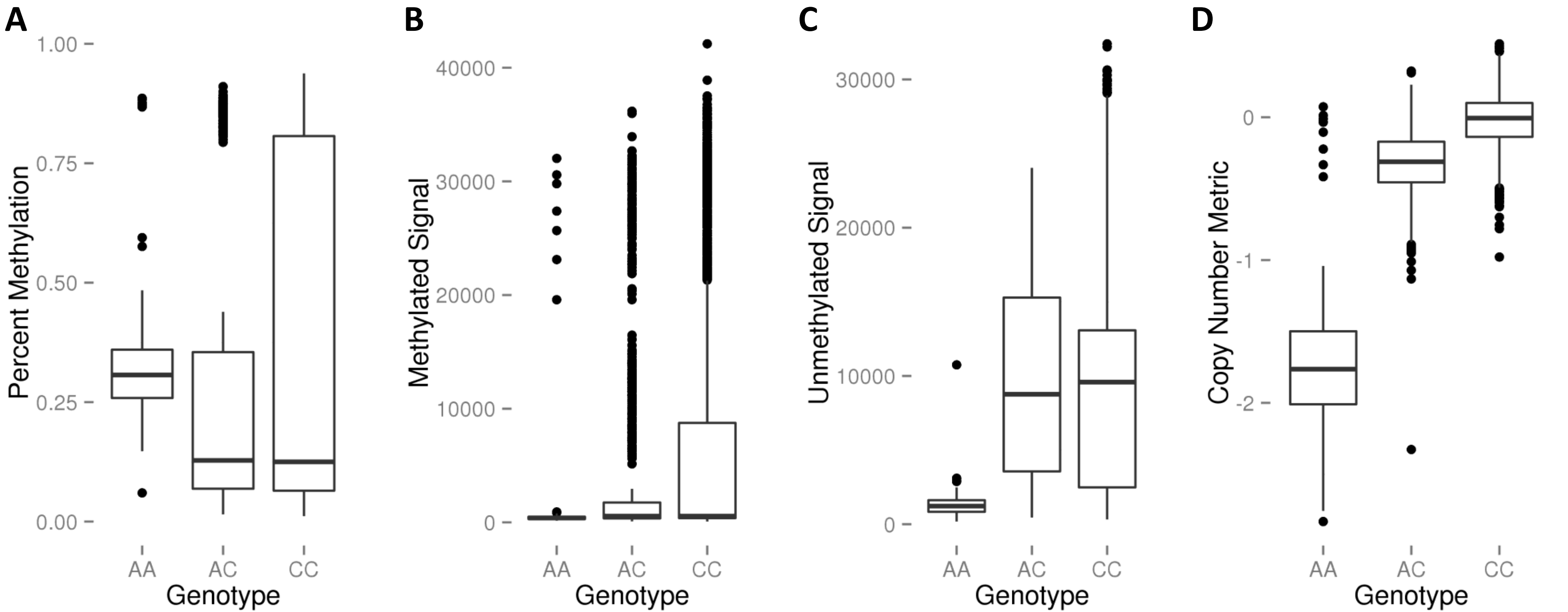

Figure S18: The effect of a A/C SNP at a negative strand C site of Type I 450k probes on percent methylation, methylated signal, unmethylated signal, and a copy number metric. 29 probes in SEED were classified in this specific scenario. **Panel A**: Percent methylation (beta value) vs. genotype. **Panel B**: Methylated signal vs genotype. **Panel C**: Unmethylated signal vs genotype. **Panel D**: Copy number metric (see Methods for calculation) vs. genotype.

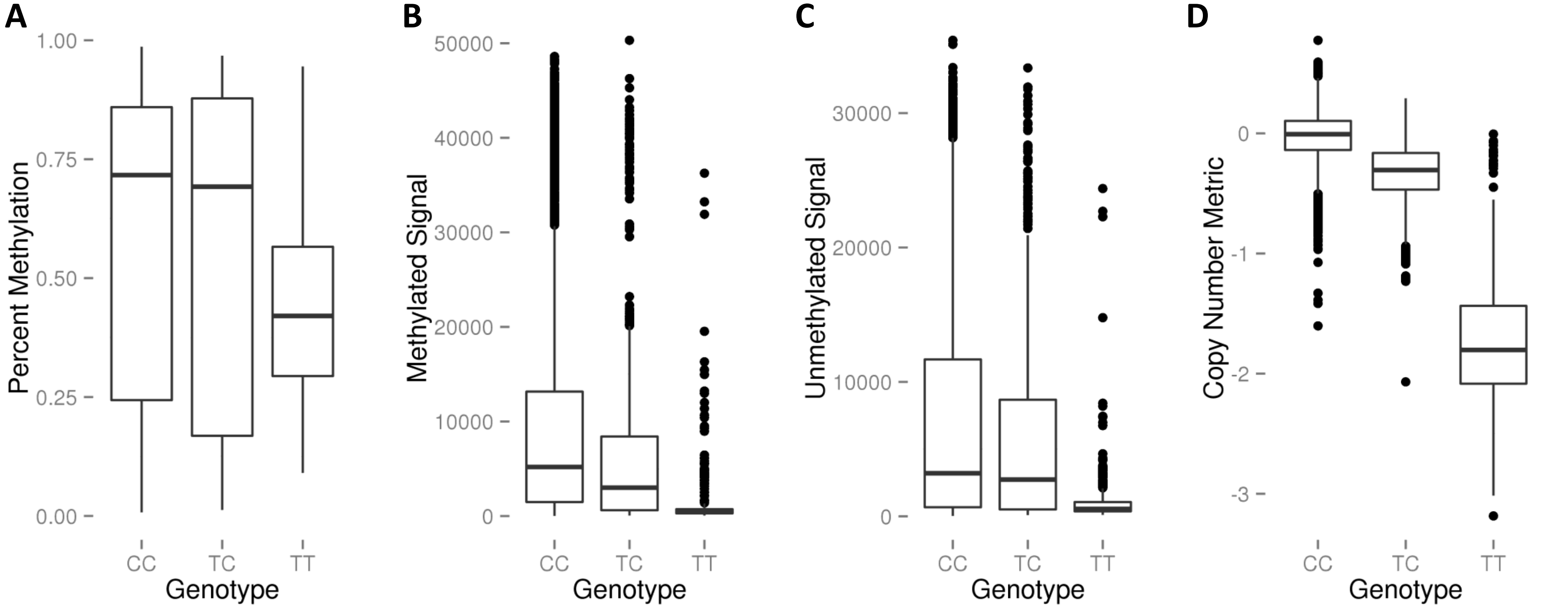

Figure S19: The effect of a T/C SNP at a negative strand C site of Type I 450k probes on percent methylation, methylated signal, unmethylated signal, and a copy number metric. 115 probes in SEED were classified in this specific scenario. **Panel A**: Percent methylation (beta value) vs. genotype. **Panel B**: Methylated signal vs genotype. **Panel C**: Unmethylated signal vs genotype. **Panel D**: Copy number metric (see Methods for calculation) vs. genotype.

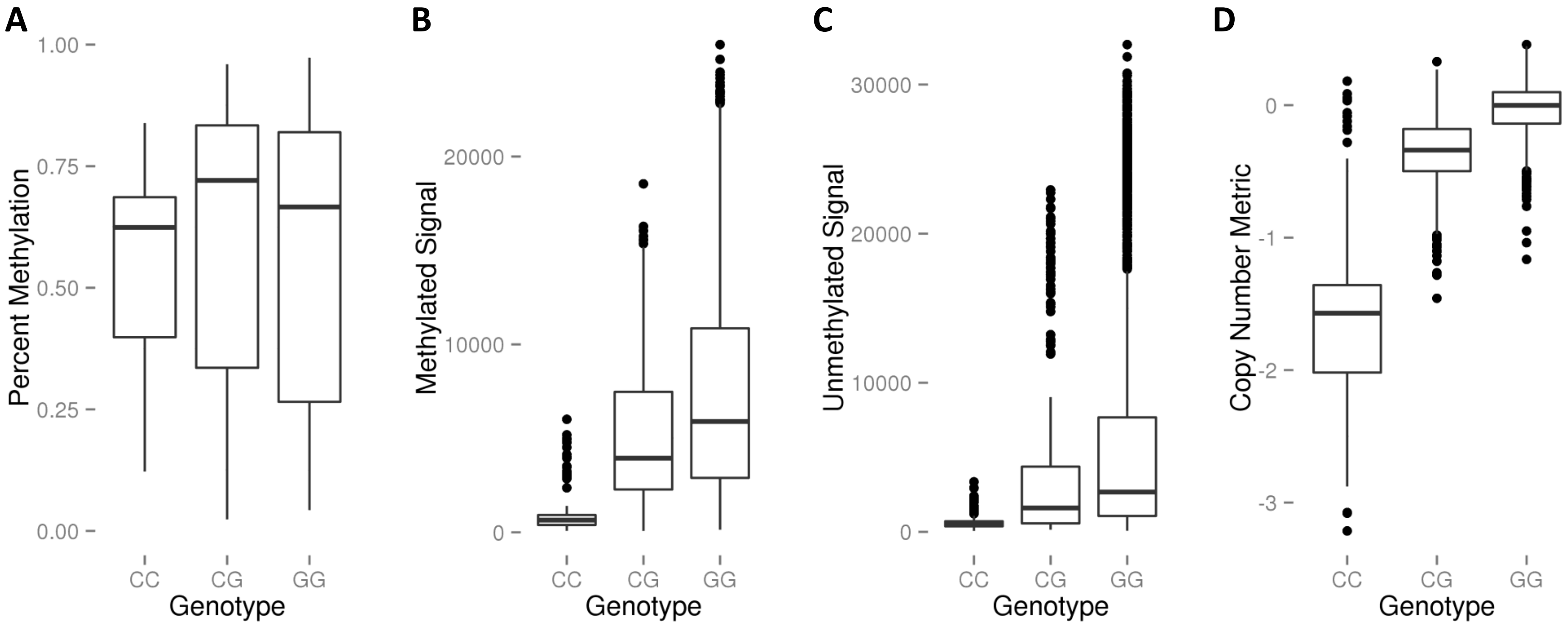

Figure S20: The effect of a C/G SNP at a positive strand G site of Type I 450k probes on percent methylation, methylated signal, unmethylated signal, and a copy number metric. 24 probes in SEED were classified in this specific scenario. **Panel A**: Percent methylation (beta value) vs. genotype. **Panel B**: Methylated signal vs genotype. **Panel C**: Unmethylated signal vs genotype. **Panel D**: Copy number metric (see Methods for calculation) vs. genotype.

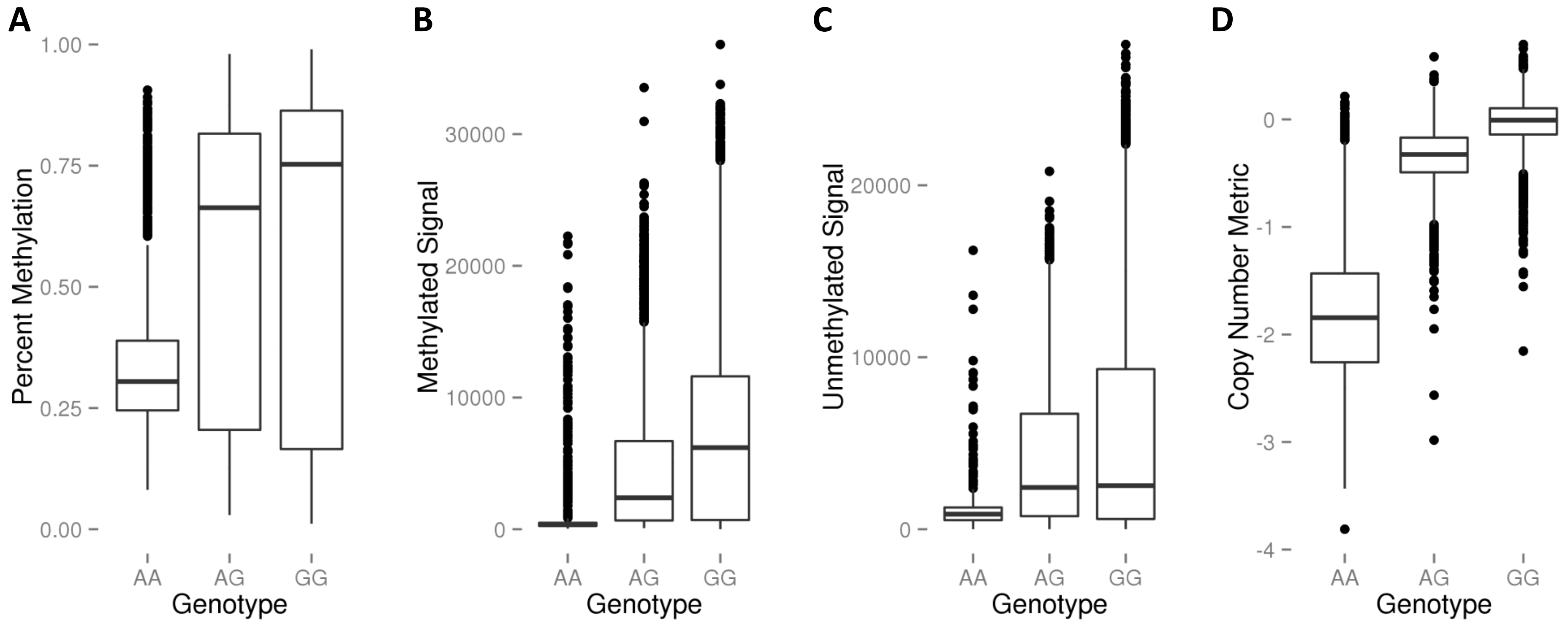

Figure S21: The effect of a A/G SNP at a positive strand G site of Type I 450k probes on percent methylation, methylated signal, unmethylated signal, and a copy number metric. 145 probes in SEED were classified in this specific scenario. **Panel A**: Percent methylation (beta value) vs. genotype. **Panel B**: Methylated signal vs genotype. **Panel C**: Unmethylated signal vs genotype. **Panel D**: Copy number metric (see Methods for calculation) vs. genotype.

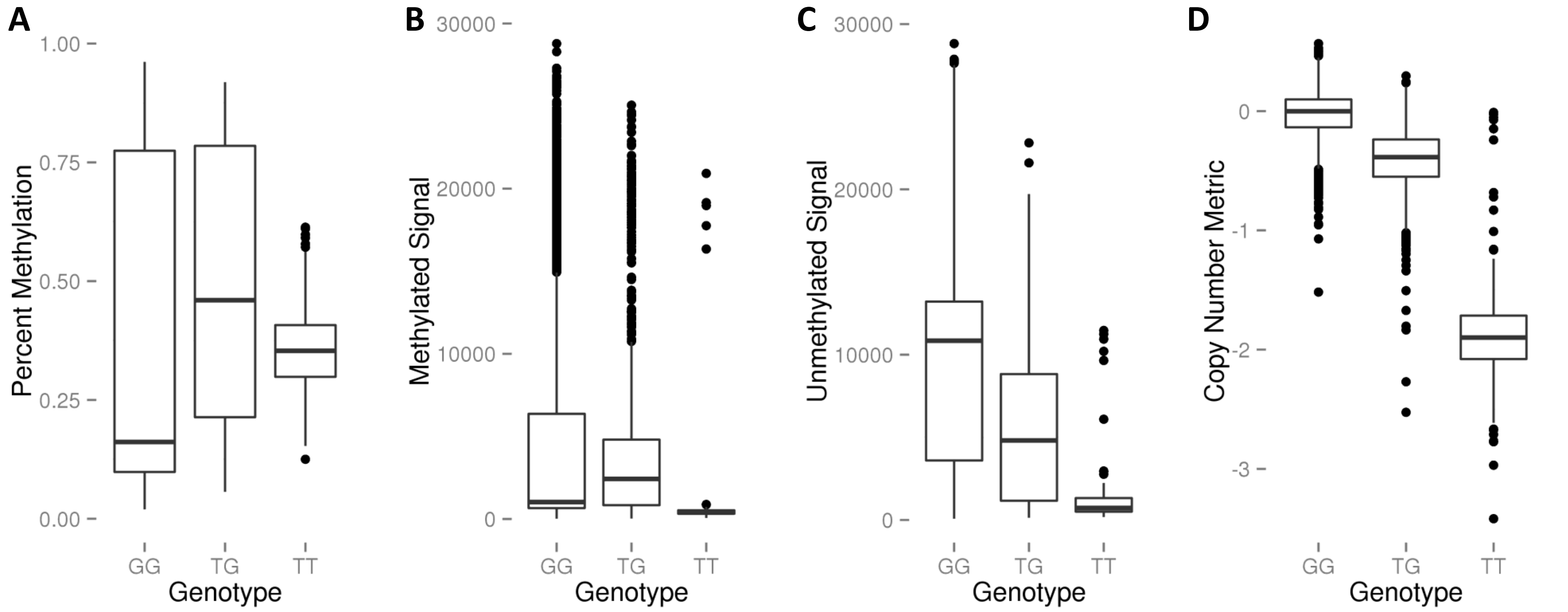

Figure S22: The effect of a T/G SNP at a positive strand G site of Type I 450k probes on percent methylation, methylated signal, unmethylated signal, and a copy number metric. 27 probes in SEED were classified in this specific scenario. **Panel A**: Percent methylation (beta value) vs. genotype. **Panel B**: Methylated signal vs genotype. **Panel C**: Unmethylated signal vs genotype. **Panel D**: Copy number metric (see Methods for calculation) vs. genotype.

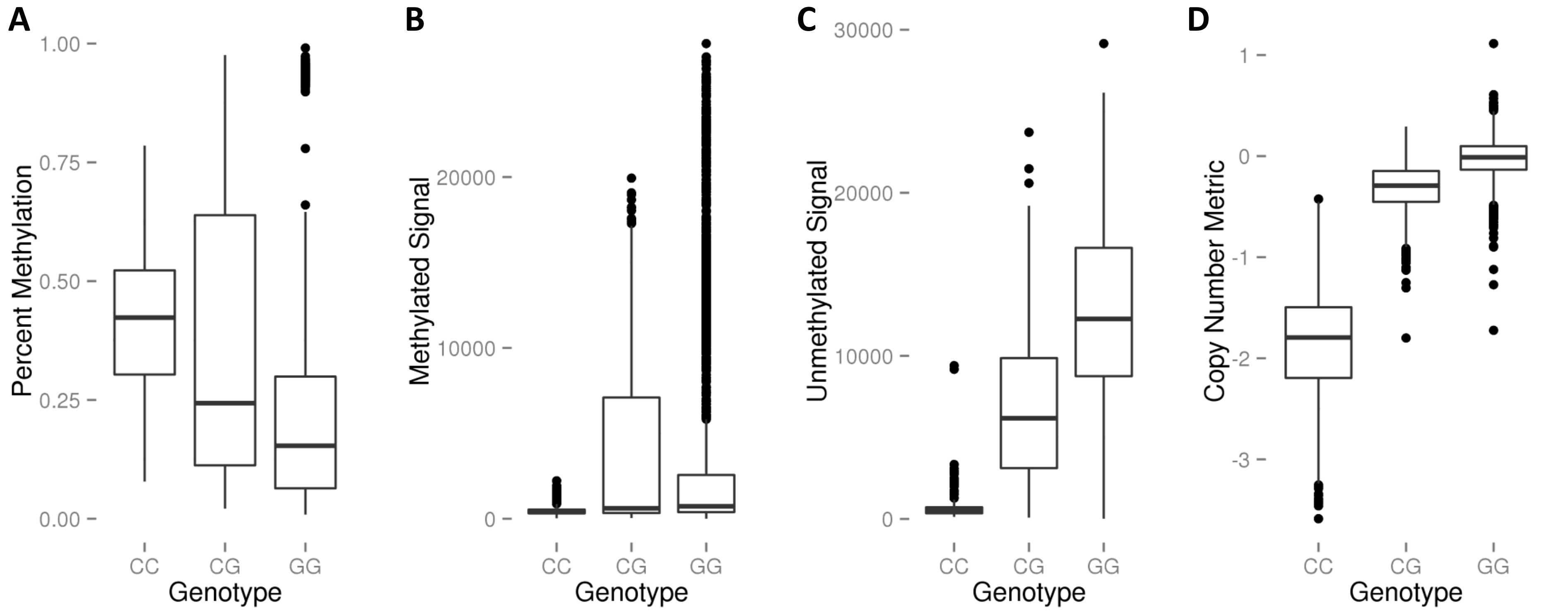

Figure S23: The effect of a C/G SNP at a negative strand G site of Type I 450k probes on percent methylation, methylated signal, unmethylated signal, and a copy number metric. 30 probes in SEED were classified in this specific scenario. **Panel A**: Percent methylation (beta value) vs. genotype. **Panel B**: Methylated signal vs genotype. **Panel C**: Unmethylated signal vs genotype. **Panel D**: Copy number metric (see Methods for calculation) vs. genotype.

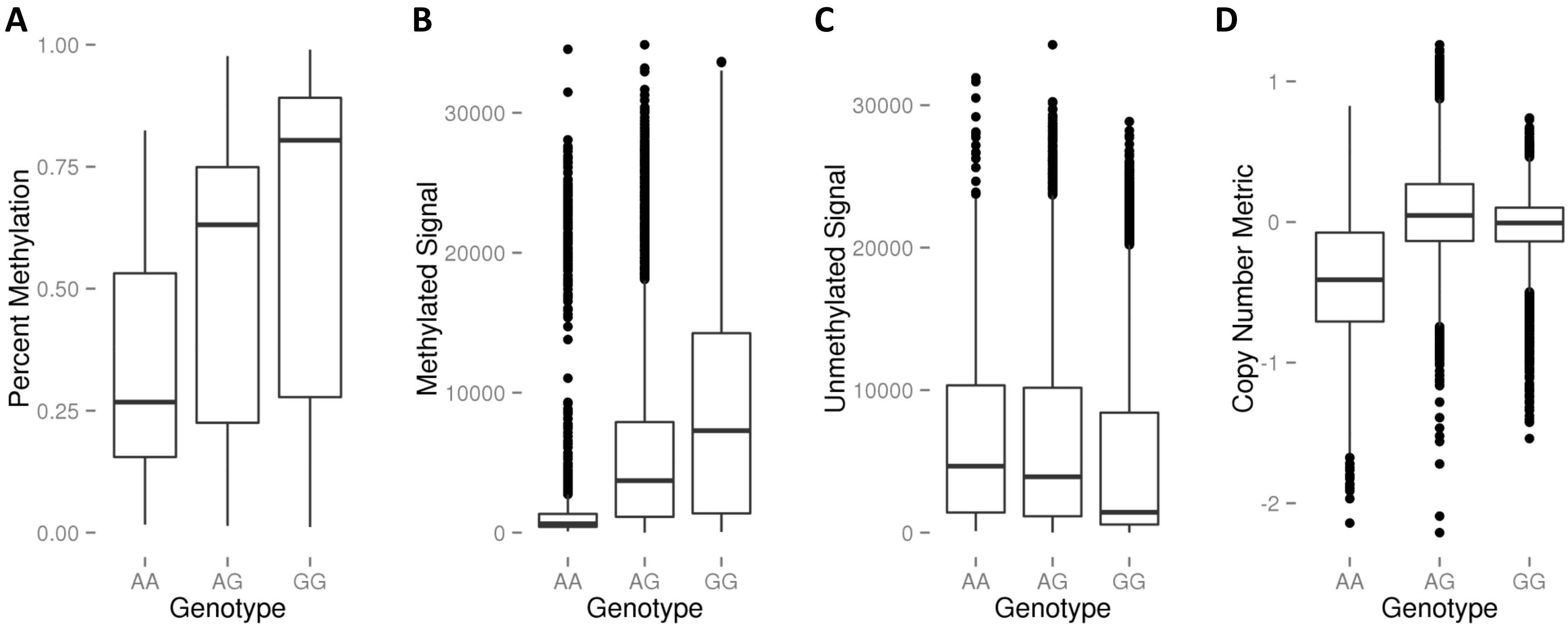

Figure S24: The effect of a A/G SNP at a negative strand G site of Type I 450k probes on percent methylation, methylated signal, unmethylated signal, and a copy number metric. 180 probes in SEED were classified in this specific scenario. **Panel A**: Percent methylation (beta value) vs. genotype. **Panel B**: Methylated signal vs genotype. **Panel C**: Unmethylated signal vs genotype. **Panel D**: Copy number metric (see Methods for calculation) vs. genotype.

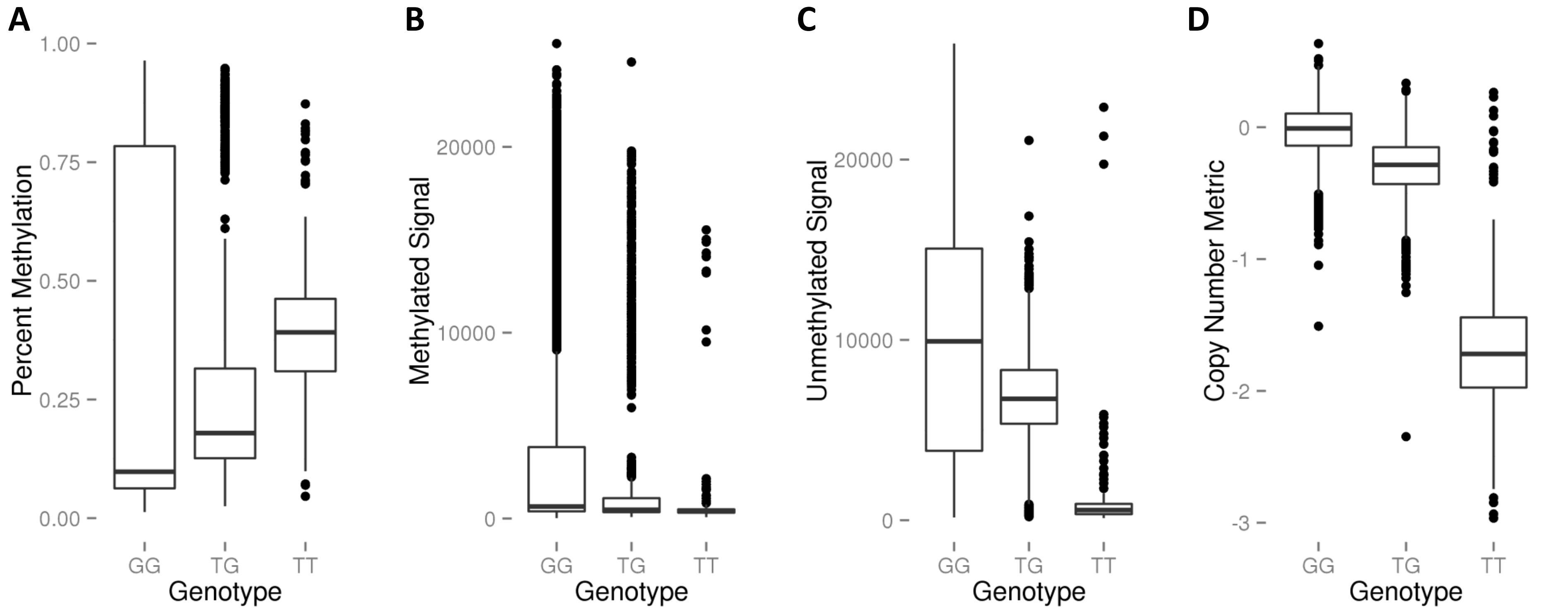

Figure S25: The effect of a T/G SNP at a negative strand G site of Type I 450k probes on percent methylation, methylated signal, unmethylated signal, and a copy number metric. 34 probes in SEED were classified in this specific scenario. **Panel A**: Percent methylation (beta value) vs. genotype. **Panel B**: Methylated signal vs genotype. **Panel C**: Unmethylated signal vs genotype. **Panel D**: Copy number metric (see Methods for calculation) vs. genotype.
