# Supplementary material for: “Gap hunting” to characterize clustered probe signals in Illumina methylation array data
Source: Epigenetics Chromatin. 2016 Dec 7;9:56. doi: 10.1186/s13072-016-0107-z (PMC5142147; doi:10.1186/s13072-016-0107-z)
Supplement: Supplementary file 6 — Additional file 6: Figures S26–S31. All remaining SBE site scenarios. Each additional scenario of a SBE site-mapping SNP delimited in Fig. 4 not including the scenario shown in Fig. 5. Each of these figures contains 4 plots, showing every combination of CpG site interrogations on the forward and reverse strand as well as which nucleotide is the reference nucleotide. [file 13072_2016_107_MOESM6_ESM.pdf]

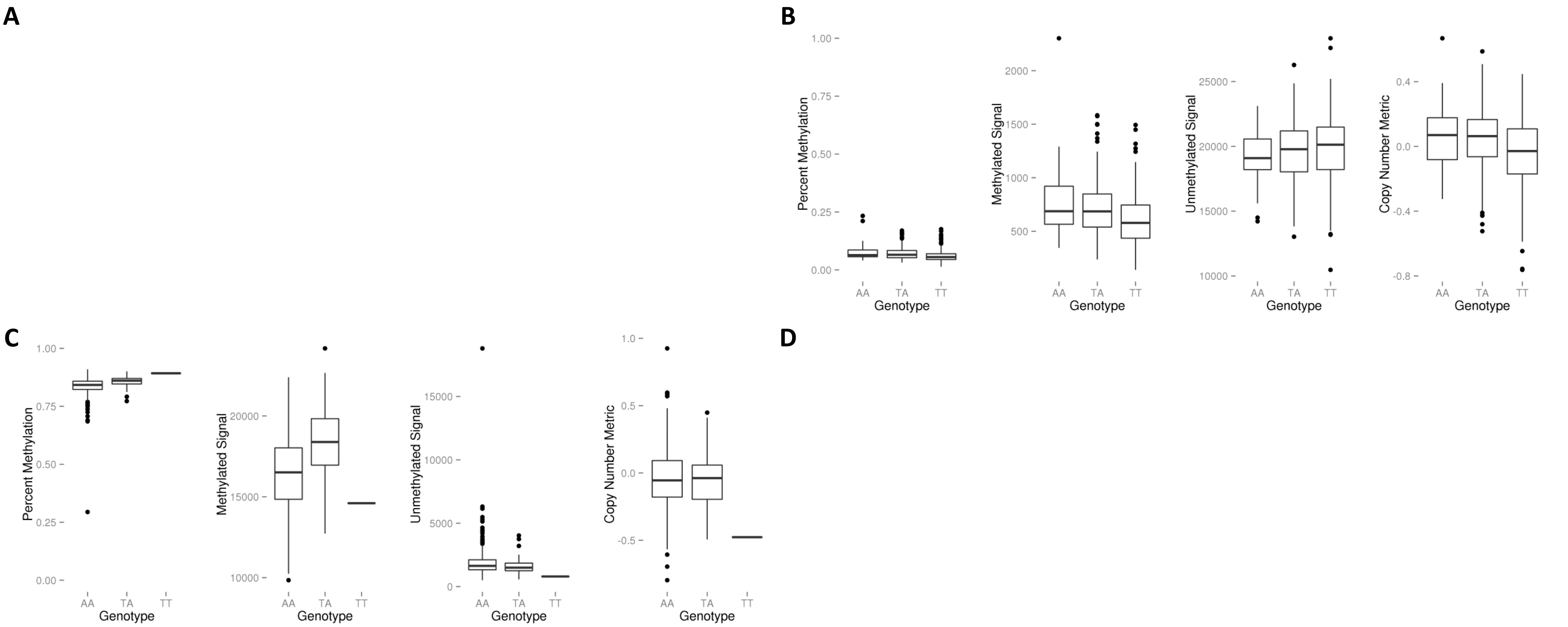

Figure S26: The effect of a T/A SNP at the SBE site of Type I probes on percent methylation, methylated signal, unmethylated signal, and a copy number metric. Panel A: Positive strand of CpG interrogation, T is reference allele (0 probes). **Panel B**: Negative strand of CpG interrogation, T is reference allele (1 probe). **Panel C**: Positive strand of CpG interrogation, A is reference allele (1 probe). **Panel D**: Negative strand of CpG interrogation, A is reference allele (0 probes).

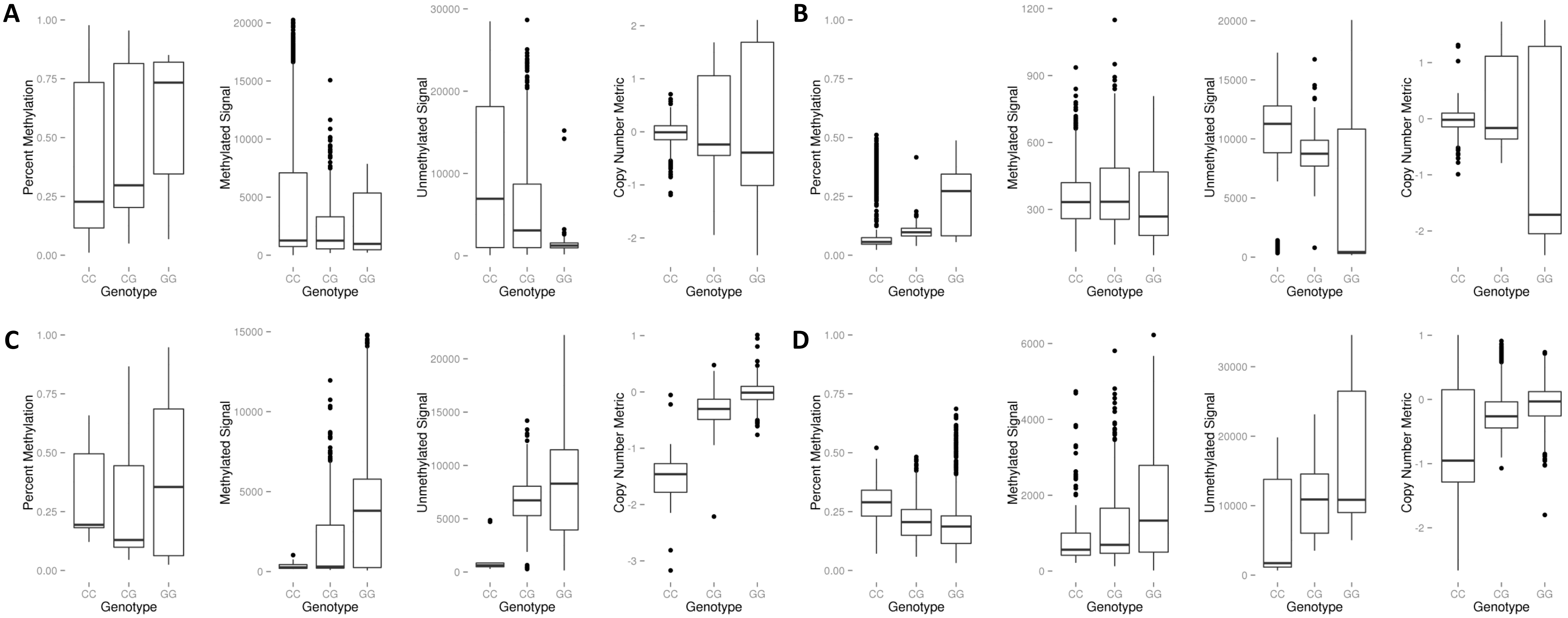

Figure S27: The effect of a C/G SNP at the SBE site of Type I probes on percent methylation, methylated signal, unmethylated signal, and a copy number metric. Panel A: Positive strand of CpG interrogation, C is reference allele (9 probes). **Panel B**: Negative strand of CpG interrogation, C is reference allele (5 probes). **Panel C**: Positive strand of CpG interrogation, G is reference allele (6 probes). **Panel D**: Negative strand of CpG interrogation, G is reference allele (5 probes).

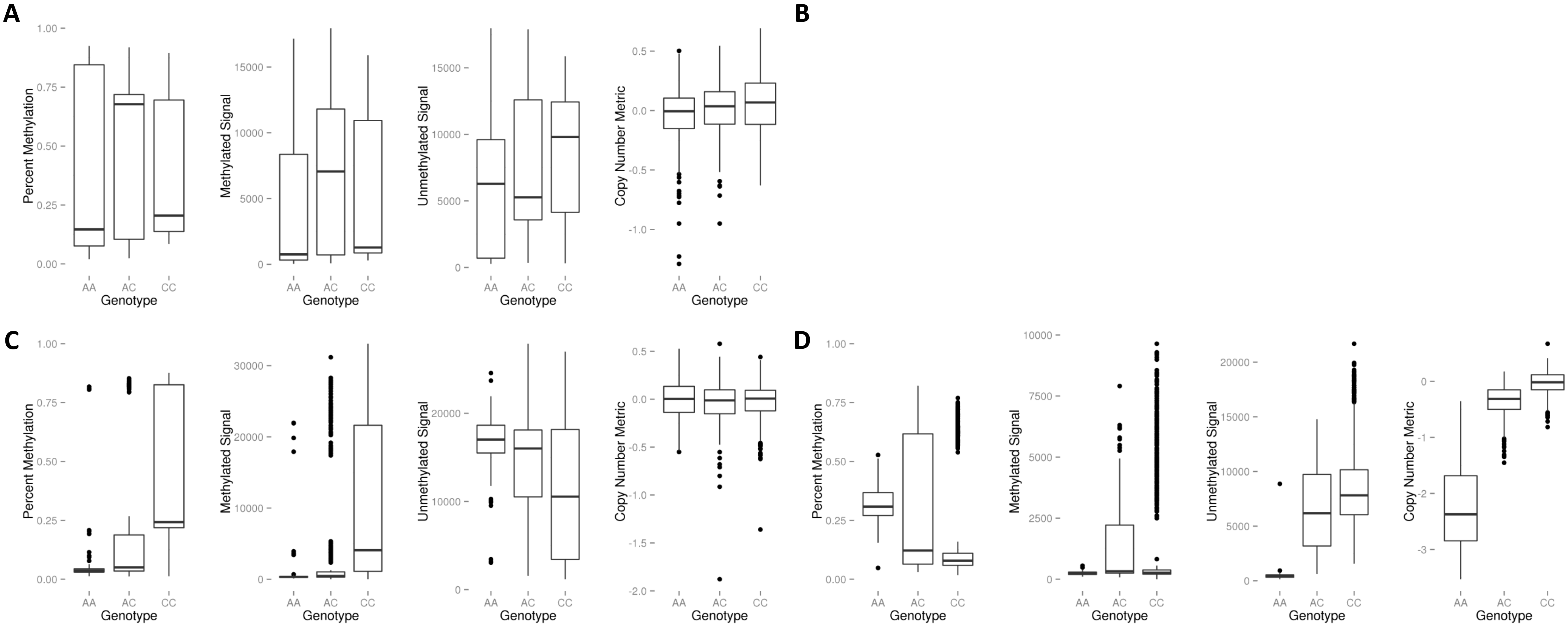

Figure S28: The effect of a A/C SNP at the SBE site of Type I probes on percent methylation, methylated signal, unmethylated signal, and a copy number metric. Panel A: Positive strand of CpG interrogation, A is reference allele (4 probes). **Panel B**: Negative strand of CpG interrogation, A is reference allele (0 probes). **Panel C**: Positive strand of CpG interrogation, C is reference allele (6 probes). **Panel D**: Negative strand of CpG interrogation, C is reference allele (7 probes).

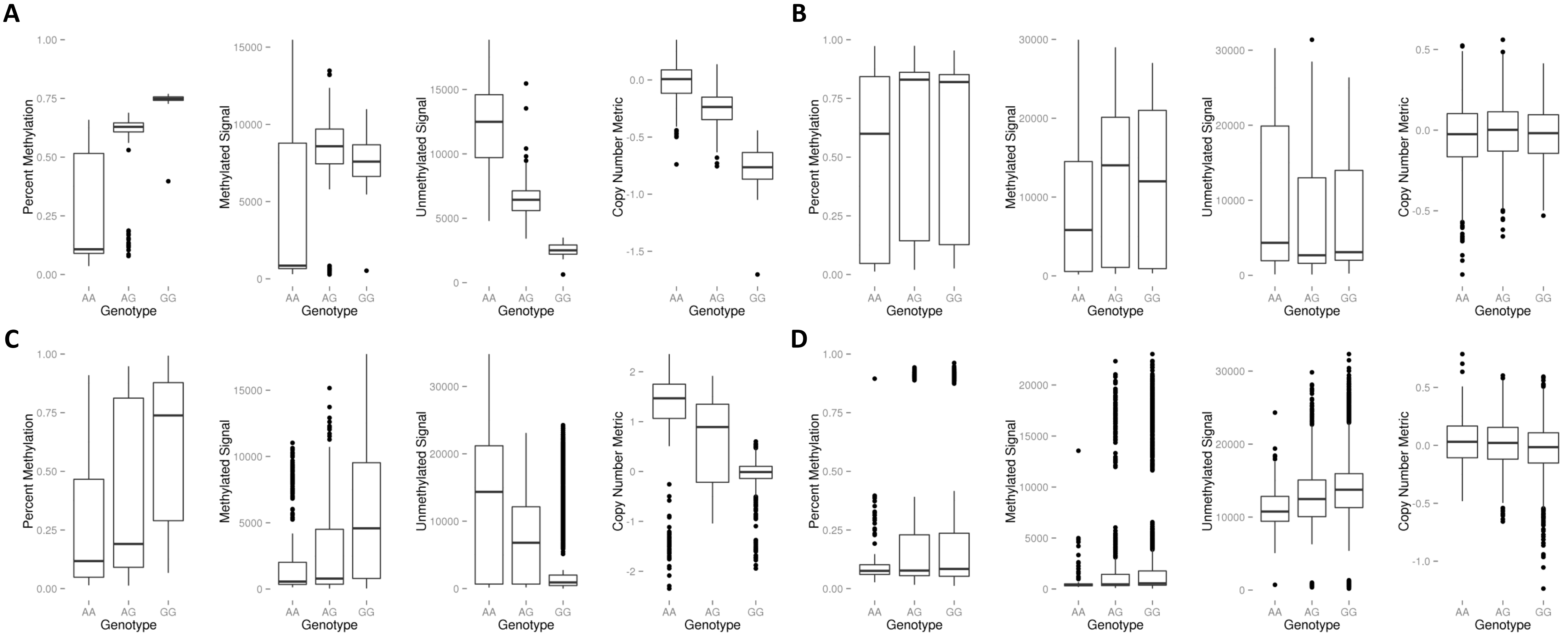

Figure S29: The effect of a A/G SNP at the SBE site of Type I probes on percent methylation, methylated signal, unmethylated signal, and a copy number metric. Panel A: Positive strand of CpG interrogation, A is reference allele (2 probes). **Panel B**: Negative strand of CpG interrogation, A is reference allele (5 probes). **Panel C**: Positive strand of CpG interrogation, G is reference allele (17 probes). **Panel D**: Negative strand of CpG interrogation, G is reference allele (11 probes).

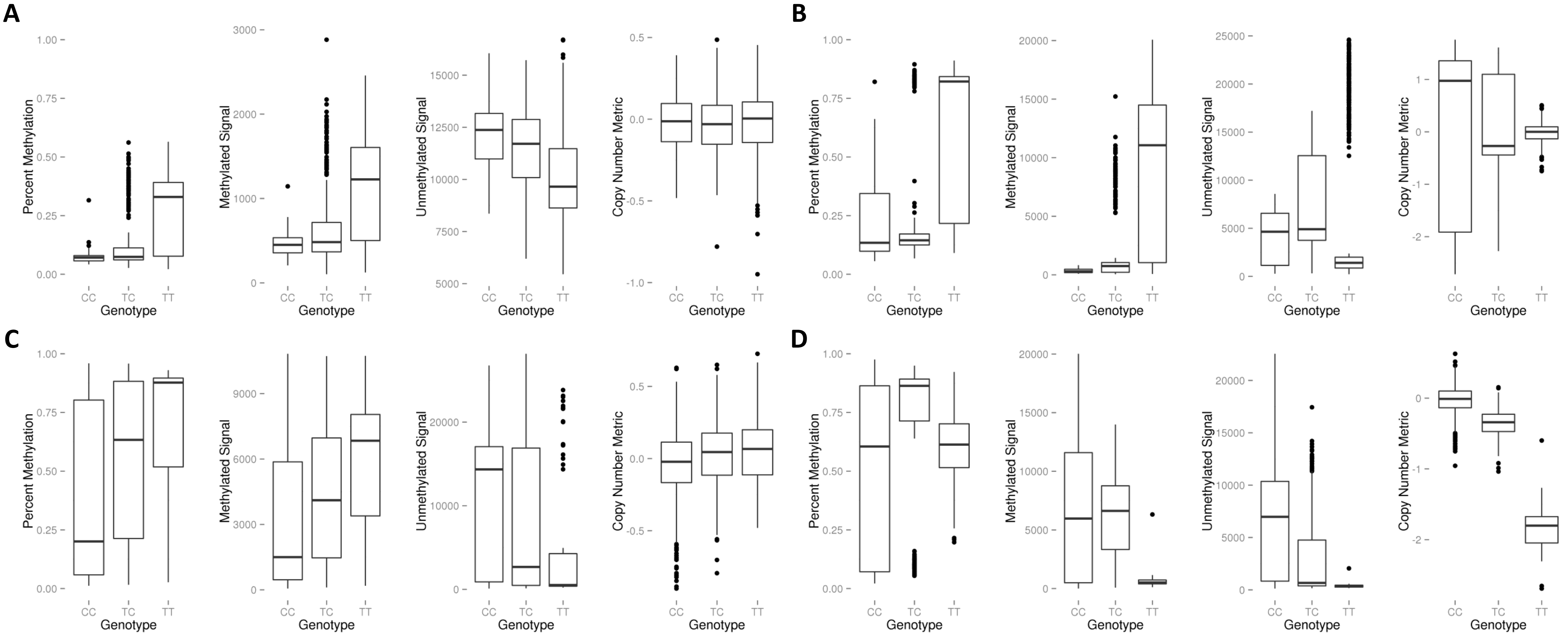

Figure S30: The effect of a T/C SNP at the SBE site of Type I probes on percent methylation, methylated signal, unmethylated signal, and a copy number metric. Panel A: Positive strand of CpG interrogation, T is reference allele (4 probes). **Panel B**: Negative strand of CpG interrogation, T is reference allele (4 probes). **Panel C**: Positive strand of CpG interrogation, C is reference allele (10 probes). **Panel D**: Negative strand of CpG interrogation, C is reference allele (10 probes).

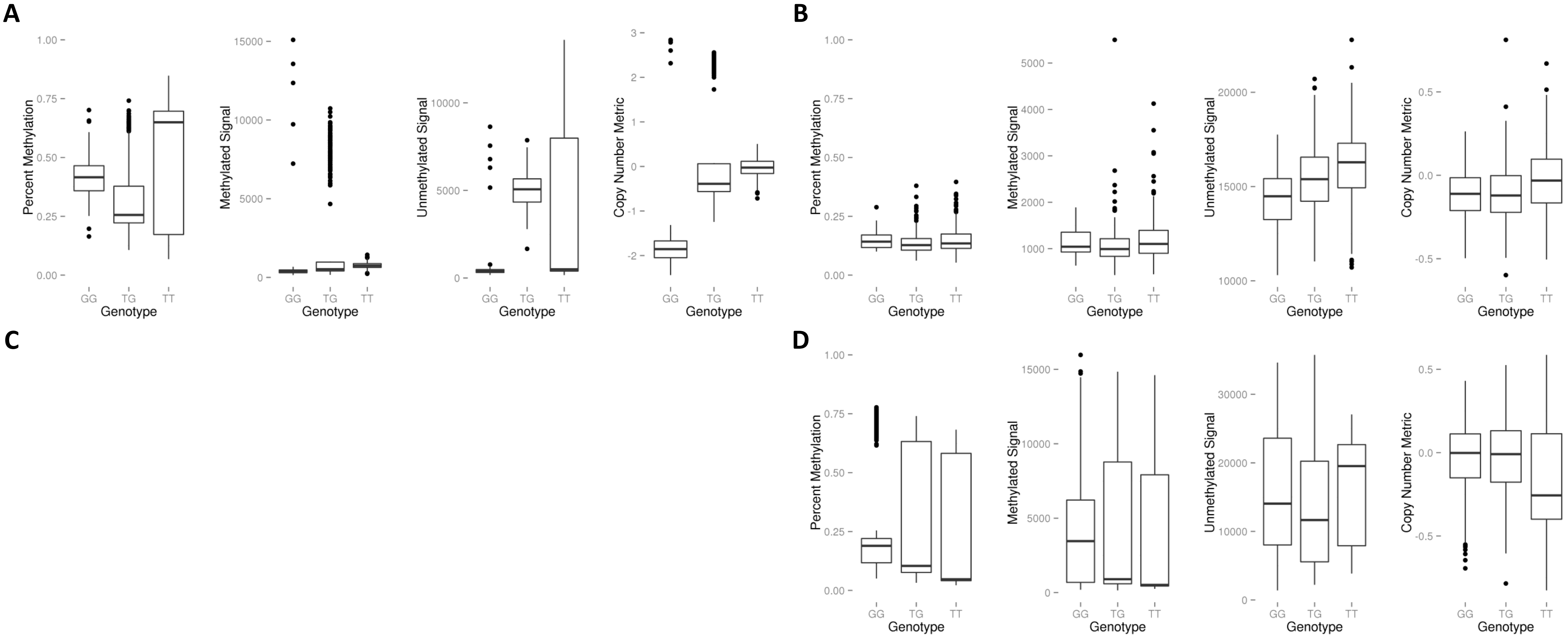

Figure S31: The effect of a T/G SNP at the SBE site of Type I probes on percent methylation, methylated signal, unmethylated signal, and a copy number metric. Panel A: Positive strand of CpG interrogation, T is reference allele (3 probes). **Panel B**: Negative strand of CpG interrogation, T is reference allele (1 probe). **Panel C**: See Figure 5. **Panel D**: Negative strand of CpG interrogation, G is reference allele (5 probes).
